# KH-like Domains in PARP9/DTX3L and PARP14 Coordinate Protein–Protein Interactions to Promote Cancer Cell Survival

**DOI:** 10.1016/j.jmb.2023.168434

**Published:** 2024-02-15

**Authors:** Hadil Saleh, Triantafillos Liloglou, Daniel J. Rigden, Jason L. Parsons, Gabrielle J. Grundy

**Affiliations:** 1 University of Liverpool, Department of Molecular and Clinical Cancer Medicine, 6 West Derby St, Liverpool L7 8TX, UK; 2 Edge Hill University, Faculty of Health, Social Care & Medicine, St Helens Road, Ormskirk, Lancashire L39 4QP, UK; 3 University of Liverpool, Department of Biochemistry, Cell and Systems Biology, Liverpool L69 7ZB, UK; 4 University of Birmingham, Institute of Cancer and Genomic Sciences, IBR West, Birmingham B15 2TT, UK

**Keywords:** ADP-ribosylation, alphafold2, protein–protein interaction, macro-PARP, regulation of expression

## Abstract

Certain members of the ADP-ribosyltransferase superfamily (ARTD or PARP enzymes) catalyse ADP-ribosylation in response to cellular stress, DNA damage and viral infection and are upregulated in various tumours. PARP9, its binding partner DTX3L and PARP14 protein levels are significantly correlated in head and neck squamous cell carcinoma (HNSCC) and other tumour types though a mechanism where PARP9/DTX3L regulates PARP14 post-transcriptionally. Depleting PARP9, DTX3L or PARP14 expression in HNSCC or HeLa cell lines decreases cell survival through a reduction of proliferation and an increase in apoptosis. A partial rescue of survival was achieved by expressing a PARP14 truncation containing a predicted eukaryotic type I KH domain. KH-like domains were also found in PARP9 and in DTX3L and contributed to protein–protein interactions between PARP9-DTX3L and PARP14-DTX3L. Homodimerization of DTX3L was also coordinated by a KH-like domain and was disrupted by site-specific mutation. Although, cell survival promoted by PARP14 did not require ADP-ribosyltransferase activity, interaction of DTX3L *in vitro* suppressed PARP14 auto-ADP-ribosylation and promoted trans-ADP-ribosylation of PARP9 and DTX3L. In summary, we characterised PARP9-DTX3L-PARP14 interactions important to pro-survival signalling in HNSCC cells, albeit in PARP14 catalytically independent fashion.

## Highlights

• PARP9 and PARP14 are lesser studied ADP-ribosyltransferases implicated in cancer cell survival.
• PARP9 and DTX3L regulate PARP14 protein levels therefore depleting PARP9, DTX3L or PARP14 mRNA results in loss of PARP14 protein and a reduction in cancer cell survival through a loss of proliferation and increase in apoptosis.
• PARP14′s promotion of cell survival does not depend on its ADP-ribosylation activity but requires a predicted KH domain in the C-terminus region that is necessary for interacting with DTX3L.
• A KH-like domain (KHL) was also predicted in PARP9 and five in DTX3L. PARP14 KHL and PARP9 KHL both interact with KHL4 of DTX3L by different means, in a tertiary complex that facilitates PARP14 trans-ADP-ribosylation of PARP9 and DTX3L *in vitro*.
• KHL1 of DTX3L forms a homodimerisation surface that allows a heterodimeric complex to form with PARP9.

## Graphical abstract

**Figure:**
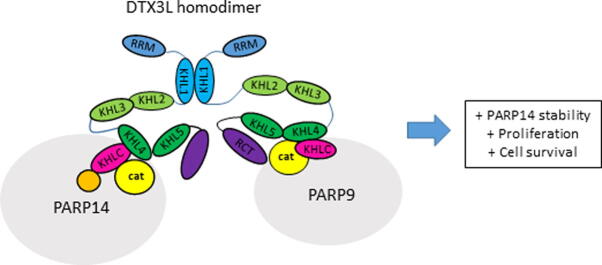

## Introduction

The ADP-ribosyltransferase superfamily (ARTD, PARP) catalyse the addition of ADP-ribose from nicotinamide dinucleotide (NAD^+^) to acceptor residues within the proteins themselves (auto-ADP-ribosylation), or onto target proteins (trans-ADP-ribosylation).^1^ All 17 family members have a catalytic domain (usually C-terminal) but differ considerably in the N-terminal domains which determine how they are activated, and which affects their function.^1^ ADP-ribosyltransferases that catalyse ADP-ribosylation in response to DNA damage (PARP1, PARP2 and PARP3) are well characterised in comparison to the remaining family members, particularly the diverse group of mono ADP-ribosyltransferases.

One subgroup of the ARTD superfamily is classified as the macro-PARPs (PARP9, PARP14 and PARP15), because each contains two or more macrodomains, modules common to several proteins that recognise or potentially hydrolyse ADP-ribose modifications.^2^, ^3^ Binding studies have characterised the two macrodomains within PARP9 as recognising poly-ADP-ribose whereas the three macrodomains of PARP14 recognise mono-ADP-ribose.^4^, ^5^, ^6^ The macro-PARPs catalyse mono-ADP-ribosylation: however, PARP9 was believed to be inactive until it was discovered that its binding partner DTX3L was necessary for ADP-ribosylation of ubiquitin.^7^, ^8^ All macro-PARPs and DTX3L form part of a cluster on chromosome 3 q21 and are interferon-stimulated genes (ISG).^9^ PARP9/DTX3L complex has been assigned anti-viral functions due to an ability to bind long double stranded RNA and ubiquitinate viral proteases.^10^, ^11^ Indeed, mono-ADP-ribosylation following IFN stimulation has been found largely attributable to PARP9.^12^ PARP14 also facilitates the IFN response to infection by promoting nuclear transport of other ISGs.^13^ PARP14 functions as co-transcriptional activator of STAT6 genes and promotes Th2 response in B-cells and can also influence STAT1 signalling in macrophages on IL-4 stimulation and thus is important for regulation of immune responses.^14^, ^15^, ^16^

Much attention has been given to pro-tumour roles of PARP9 and PARP14 in a variety of cancer types, particularly as some tumours have elevated levels of expression of transcript and protein. PARP9 is also known as BAL1 (B aggressive lymphoma-1 protein), a risk factor whose overexpression prevented IFN-induced apoptosis in aggressive diffuse large B-cell lymphoma DLBCL.^17^ High PARP9 transcript levels have been detected in glioma, prostate, breast and other solid tumours,^7^, ^18^, ^19^, ^20^ whereas PARP14 overexpression has been reported in multiple myeloma, metastatic prostate cancer, drug resistant pancreatic cancer and hepatocellular carcinoma.^19^, ^21^, ^22^, ^23^ PARP14 has furthermore been demonstrated to promote pro-survival pathways including NF-κB signalling, inhibition of pro-apoptotic factor JNK1 and G1/S cell cycle progression through cyclin D1 expression,^21^, ^22^, ^23^, ^24^, ^25^ whereas PARP9 has pro-survival and pro-metastatic roles in DLBLC and breast cancer, respectively.^17^, ^20^ In one report, PARP14 and PARP9/DTX3L were found to form a tertiary complex and to co-operate to provide anti-apoptotic and pro-survival properties.^19^

We have explored further the molecular requirements of PARP9/DTX3L and PARP14 mediated survival using head and neck squamous cell carcinoma (HNSCC) and HeLa cell lines, which have increased PARP9/DTX3L and PARP14 expression. We have found that PARP14 protein expression is correlated and regulated by PARP9 and DTX3L not through at transcription or by proteasomal or lysosomal degradation post-translationally. All three proteins effected cell survival by reducing colony formation and proliferation when depleted by siRNA. Colony formation can be partially rescued by overexpressing a C-terminal PARP14 fragment containing an uncharacterised domain. This domain was identified to be a K-homology domain (KH) through Alphafold2 (AF2) and FoldSeek prediction tools and found similar domains PARP9 and DTX3L. Canonical KH domains are involved in RNA/DNA binding, but we demonstrate that certain PARP9/DTX3L and PARP14 KH-like domains were involved in protein–protein interactions. We mapped and modelled the interactions of the tertiary complex and verified the interface by disrupting interactions with mutating key residues. PARP14 mediated survival did not require PARP14 catalytic activity and the effect of DTX3L binding to PARP14 suppressed auto-ADP-ribosylation.

## Results

### Stability of PARP14 is regulated by PARP9-DTX3L independent of proteasomal or lysosomal degradation

**Figure 1:**
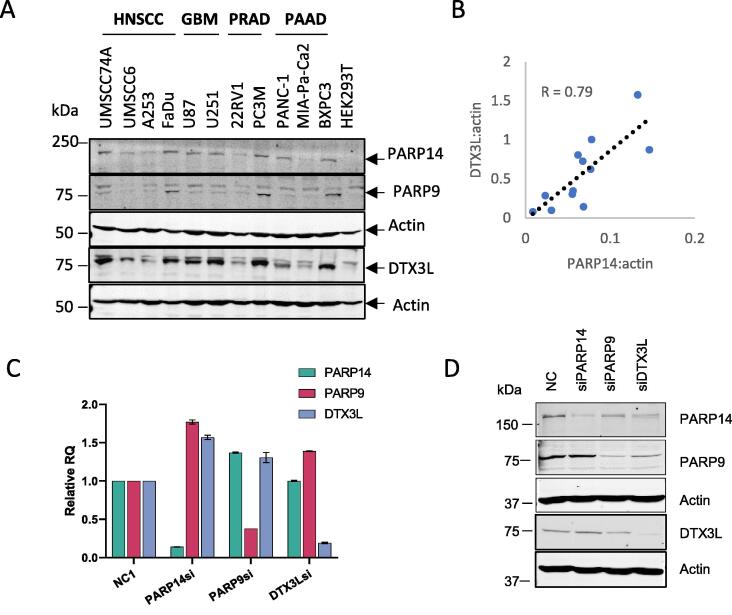
PARP9/DTX3L regulates of PARP14 post-transcriptionally. A) Immunoblot analysis of a panel of cell-lines, including HNSCC (head and neck squamous cell carcinoma), GBM (glioblastoma multiforme), PRAD (Prostate adenocarcinoma) and PAAD Pancreatic adenocarcinoma. B) Correlation of PARP14 and DTX3L protein levels in cell lines, showing Pearson correlation R. C) RT-qPCR analysis of total mRNA using gene-specific primers for PARP9, PARP14 and DTX3L in cells transfected with siRNAs specific to each respective gene or NC1: negative control. The relative expression levels were quantified using the ΔΔCt method and presented as RQ. The expression values were normalised to ACTB as the reference gene. The data is presented as the mean values, with the error bars representing the standard deviation (SD). D) Immunoblot analysis of PARP14, PARP9 and DTX3L in FaDu cells following 48 hr transfection with siRNAs specific to each respective gene or NC1. Actin was used as a loading control.

Our initial aim was to explore the expression of pro-tumour ADP-ribosyltransferases PARP14 and PARP9/DTX3L particularly in HNSCC, which are characteristically resistant to treatment and PARP9/14 mediated survival might represent a mechanism for increased survival. We compared protein expression of PARP14 and its binding partner, DTX3L, proteins in a panel of different cell lines, (HNSCC; Glioblastoma GBM; Prostate Adenocarcinoma PRAD; and Pancreatic Adenocarcinoma PAAC). PARP9 and PARP14 protein levels were variable among the cell lines but PARP14 correlated well with DTX3L protein with a Pearson correlation *R* = 0.79 (Figure 1A,B). Further examination of PARP9 and PARP14 protein expression in HNSCC showed no association with p53 or HPV status (Figure S1A).

The roles of PARP9, DTX3L and PARP14 in HNSCC were examined by depletion using siRNAs. Double stranded siRNAs were efficient in knocking-down the respective mRNA levels in several cell lines, as determined by qPCR, including FaDu (<20% of negative control NC siRNA levels, Figure 1C), UMSCC74A and HeLa (Figure S1B). When the protein levels in FaDu were assessed by immunoblotting, it was observed that siRNA to PARP9 (siPARP9) also led to a reduction in DTX3L and PARP14 proteins (<60% of NC, Figure 1D). Likewise, siRNA targeting DTX3L (siDTX3L) also reduced PARP9 (30% of NC) and PARP14 (45% of NC) protein levels. However, siRNA to PARP14 had no effect on either PARP9 or DTX3L protein levels. This phenomenon was also observed in UMSCC74A, UMSCC17A (HNSCC), HeLa and BXPC3 (PAAC) cells (Figure S1B, S3C). No reduction of PARP14 mRNA was detected in the siPARP9 or siDTX3L treated FaDu cells (Figure 1C) or HeLa and UMSCC74A (Figure S1C), ruling out regulation at the transcription level. Thus, PARP9 and DTX3L proteins are required to maintain the stability of PARP14 protein levels enforcing PARP9/DTX3L-PARP14 protein level correlation.

**Figure 2:**
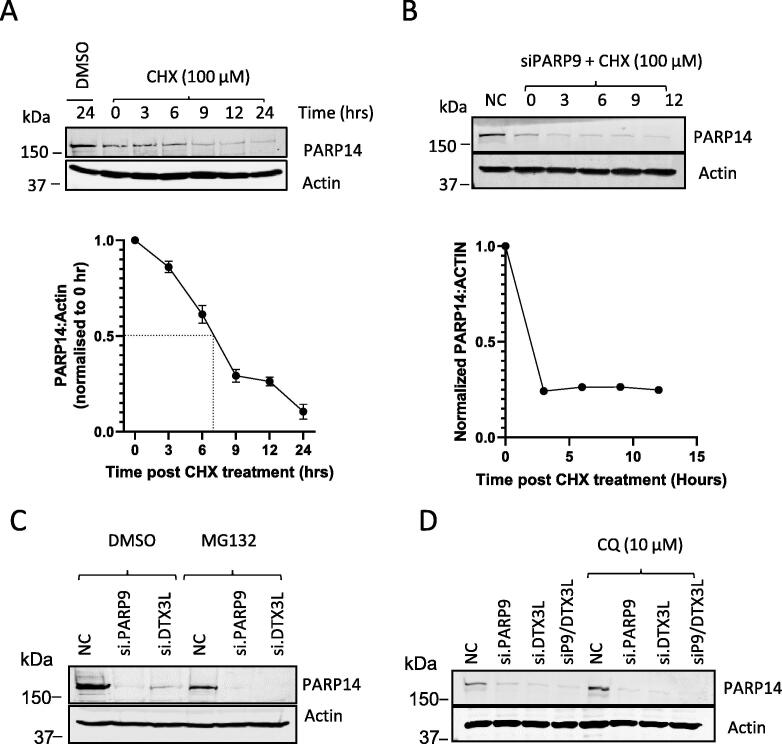
PARP14 is removed rapidly in the absence of PARP9/DTX3L but independently of the proteasomal or lysosomal degradation. A) Immunoblot analysis and quantification of PARP14 degradation in UMSCC74A after cycloheximide (100 μM) treatment. B) Immunoblot analysis and quantification of PARP14 degradation in siPARP9-transfected cells following CHX (100 μM). C) Immunoblot analysis of PARP14 in NC1, siPARP9 and siDTX3L treated UMSCC74A cells in the presence of proteasomal inhibitor, MG132 (10 μM) or DMSO control. D) Immunoblot analysis of PARP14 in NC1, siPARP9, siDTX3L and siPARP9/siDTX3L UMSCC74A cells in the presence of CQ (10 μM).

PARP9 is known to form a stable complex with DTX3L, so that PARP9 levels are reduced with siDTX3L and *vice versa*. We determined that PARP14 protein had a half-life of 8 h in UMSCC74A cells by blocking *de novo* protein synthesis with cycloheximide (Figure 2A). In PARP9 siRNA depleted cells, the effect of cycloheximide was very rapid with PARP14 levels displaying a half-life of ∼1.5 h (Figure 2B). In the absence of PARP9/DTX3L complex it was envisaged that PARP14 might be targeted for proteasomal degradation. Proteasome inhibitor MG132 did not recover PARP14 protein levels in PARP9/DTX3L depleted cells (Figure 2C), suggesting PARP14 is not regulated by proteasomal degradation. Furthermore, the use of chloroquine, a lysosomal inhibitor, increased the amount of PARP14 in UMSCC74A cells (approximately two-fold) but failed to rescue PARP14 from degradation during knockdown of PARP9 and DTX3L (Figure 2D). This demonstrates that a fraction of PARP14 is channelled into lysosomes for degradation but does not account for the decreased PARP14 in the absence of PARP9 and DTX3L.

PARP14 is pan-cellular and we examined whether PARP9 depletion effect cytoplasmic or nuclear pools or effected localisation. Cellular fractionation experiments show PARP14 to be largely nuclear under transfection conditions and siPARP9 or siDTX3L reduces PARP14 in both compartments in HeLa and FaDu cells (Figure S2A).

### Depletion of PARP9-DTX3L and PARP14 reduces survival and proliferation in cell lines independent of PARP14 catalytic activity but dependent on a region in the C-terminus

**Figure 3:**
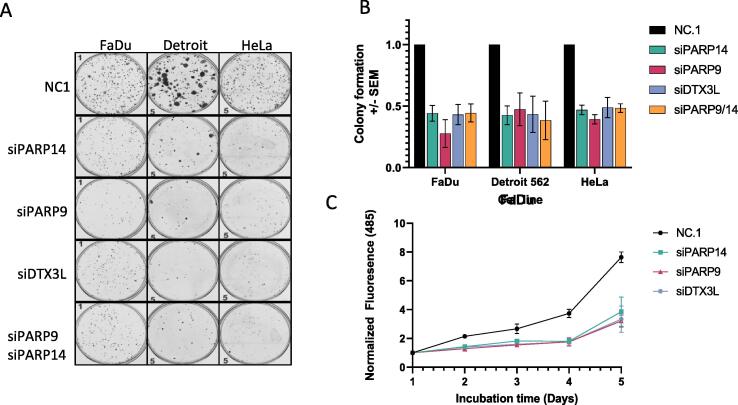
Reduction of colony formation and proliferation by PARP14, PARP9 and DTX3L depletion partial rescue with C-terminal PARP14 truncation. A) Representative images of the reduction of colony formation following siPARP9, siDTX3L, siPARP9 and double knock-down treatment relative to NC siRNA. Each pool contained 3 siRNAs. B) Normalised colony formation values from >3 independent experiments were averaged. C) Line plot of CyQUANT cell proliferation assay showing slower proliferation of PARP14, PARP9 and DTX3L depleted FaDu cells compared to NC1 cells.

Pro-survival properties of PARP9 and PARP14 were assessed by the effect of PARP9 and PARP14 depletion on colony formation in a panel of HNSCC cell lines and HeLa that possess high PARP14 expression (Figure 1A, S1A). Depletion of either PARP9, PARP14 or DTX3L with siRNA consistently decreased cell survival to 40–60% compared to a negative control siRNA in HeLa, FaDu, Detroit 562 (Figure 3A,B), A253 and PAAC cell line BXPC3 (Figure S3A,D). A smaller, but significant, reduction in colony number was observed in cell lines with low PARP14 expression (UMSCC6 and UMSCC17A, 70–90% of NC, Figure S3A). A double knock-down of PARP9 and PARP14 did not produce an additive effect on reducing colony number, suggesting that the proteins function together in the survival pathway (Figure 3A,B). Since reduction of PARP14 is also caused by siPARP9, it is plausible that the survival effect is dependent on PARP14.

A proliferation assay was conducted over 5 days following siRNA knockdown, demonstrating that depletion of PARP9, DTX3L and PARP14 significantly reduced growth rate over the time course (Figure 3C) and suggesting cell proliferation was impacted. An examination of cell cycle profiles revealed that PARP9, DTX3L or PARP14 did not significantly affect cell cycle 48 h following knock-down with siRNA in HeLa or FaDu, thus not arresting in G1 or G2 (Figure S2B,C). In previous reports, PARP14 suppressed apoptosis by inhibiting JNK1 phosphorylation in hepatocellular carcinoma and multiple myeloma cells.^21^, ^23^ Indeed, we observed a small but reproducible increase in JNK phosphorylation in FaDu and HeLa when PARP14, PARP9 or DTX3L was knocked down (1.2–1.9-fold increase Figure S4A). Furthermore, we detected an increase in cleaved PARP1 as a marker of apoptosis in knocked down cells (1.2–1.6-fold increase, Figure S4B). In cells with lower PARP14 (UMSCC6 and UMSCC17A), the amount of cleaved PARP1 was also low, correlating with the mild survival phenotype of the knockdowns (Figure S4C). Thus, the decrease in survival in the absence of PARP9/DTX3L/PARP14 is a consequence of a decrease in proliferation and an increase in apoptosis.

**Figure 4:**
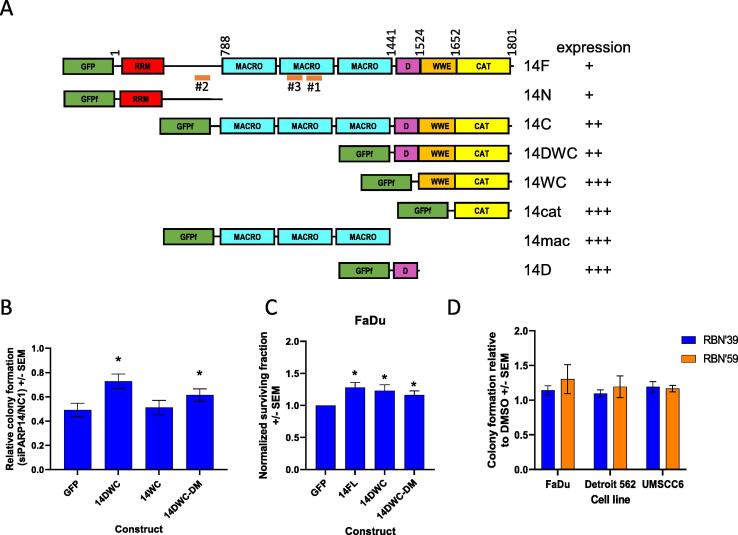
Partial rescue of colony formation with PARP14 C-terminal fragment, independent of ADP-ribosylation activity. A) Schematic representation of the primary structure of the PARP14 including macrodomains (MACRO), catalytic domain (CAT), WWE domain and a conserved region (D) and RNA recognition motifs (RRM). GFP fusions with and without a FLAG epitope (f). B) Knock-down of PARP14 using siPARP14#2 gave 50% reduction in colony formation. Colony formation of overexpression of GFP and PARP14 truncations was compared relative to overexpression in NC siRNA treated HeLa cells. The ratio was averaged over > 3 experiments, and a t-test was performed using GFP values as a control array and significance p < 0.05 is indicated by *. C) Colony formation assay using cells transfected with plasmid DNA expressing GFP tagged PARP14 in FaDu cells. Increased colony formation was assessed by t-test. D) Comparative colony formation in the presence of 1 μM RBN250039 (RBN’39, control compound) and RBN012759 (RBN’59, PARP14 catalytic inhibitor). Values are normalised to colonies 0.1% DMSO control, *n* = 3.

Attempts at rescuing cell survival through overexpression of PARP14 were complicated by poor expression of full length and most truncated versions of PARP14 in HeLa and FaDu cell lines. Expression of GFP-tagged constructs in HeLa (Figure 4A, S5A) showed that C-terminal truncations fused to GFP that contained the catalytic domain (14cat) and WWE (14WC) expressed well. However, the addition of a conserved region (‘D’) between the macrodomains and the WWE domain (14DWC) reduced expression, despite 14D also being well expressed on its own, and did not change cellular localisation (Figure S5B).

In HeLa cells, the overexpression of WWE-CAT (14WC) and 14cat truncations were unable to rescue cell survival by siPARP14#2 (which targets an N-terminal region of PARP14), whereas the inclusion of the region ‘D’ between macrodomains and WWE-CAT (14DWC) could partially rescue survival to 75% of the negative control despite lower expression (*p* = 0.013, Figure 4B). Overexpression of 14DWC in could also increase survival in FaDu cells (Figure 4C).

The contribution of PARP14′s ADP-ribosyltransferase activity to cell survival was also assessed using a selective and potent PARP14 catalytic inhibitors, (RBN012759).^26^ No difference in survival of FaDu, Detroit 562 or UMSCC6 cells was observed with this inhibitor even though it was effective in cells (Figure 4D, Figure S5A,B). A double mutation in the catalytic domain in the DWC construct was included in the complementation assay, and again a significant partial rescue of survival was seen (68%, *p* = 0.048, Figure 4B) and increased survival in FaDu (Figure 4C). Therefore, the region of unknown structure referred to as 14D appears to be important for influencing PARP14 protein levels and the survival phenotype of PARP14.

### A conserved domain in PARP9 and PARP14 adopts a predicted type I KH-like fold (KHL)

**Figure 5:**
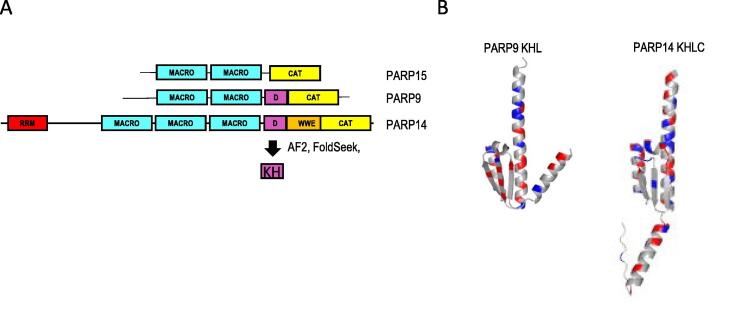
KH domains in the C-terminal regions of PARP14 and PARP9. A) Domains of macro-PARPs PARP15, PARP9 and PARP14. The intervening domain D is predicted to be a single KH domain by FoldSeek comparison of the AF2 structure with PDB database (see also Table 1). B) AF2 structure of PARP9 KH-like domain and PARP14 KH-like domain where red indicates acidic residues and blue, basic amino acids.

The region that influenced PARP14 stability and survival is ∼100 amino acids in length and is highly conserved between species (Figure S7A). Interestingly, a similar region was found in PARP9 between the macrodomains and catalytic domain, but not in the other macro-PARP family member, PARP15 (Figure 5A, Figure S7B). Secondary structure predictions for PARP9 and PARP14 gave a similar pattern of alpha and beta structures (β-α-β-α-α-β-β-α) (Figure S8A). Alphafold2 (AF2) was used to fold PARP9 and the C-terminal half of PARP14, and the resulting structures were used to find the closest structural relatives in FoldSeek. The regions corresponding to 14D and 9D were identified as eukaryotic type I KH domains typically found in RNA binding proteins. The closest matches included FUBP1 (probability 0.9, PDB:6Y2D) and Nova1 (probability 0.97, PDB:2ANR), respectively (Table 1, Figure S9).

**Table 1:** FoldSeek identification of domains (top hits).

Domain	**FoldSeek closest structural relative** PDB title protein (aligned residues)	Probability	%ID	Designation
PARP14D	6Y2D FUBP1	0.90	14.0	PARP14 KHL-C
PARP9D	2ANR Nova1	0.97	12.0	PARP9 KHL-C
DTX3L D1 (1–95)	1OIA U1Arnp (5–91)	0.98	15.2	DTX3L RRM
DTX3L D2 (130–200)	N/A	-	-	DTX3L KHL-1
DTX3L D3 (230–365)	1X4N FUSE BP1 (221–300)	0.80	16.6	DTX3L KHL2-KHL3
DTX3L D4 (366–540)	1ZTG α polyC BP (377–433) 6GY4 BICC1 (448–509)	0.96 0.95	12.3 15.0	DTX3L KHL4-KHL5

The PARP9 and PARP14 KH domains deviated from the typical KH structure with an additional β-α preceding the KH ‘minimal region’ and the key RNA-binding residues, a GXXG motif were not present. Furthermore, they had high acidic content not conducive to electrostatic interactions with RNA/DNA (AF2 structures are presented in Figure 5B). Therefore, we designated these domains as KH-like (KHL) and it was considered possible that instead of nucleic acid binding, this domain is responsible for protein interactions e.g. DTX3L.

### The C-terminal KHL domain of PARP14 interacts with a KHL domain in DTX3L

**Figure 6:**
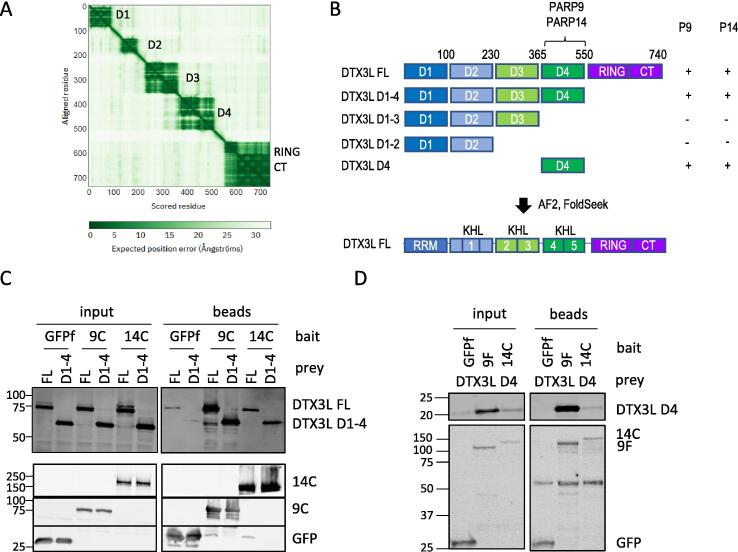
DTX3L domains and interactions with PARP9 and PARP14. A) Predicted aligned error (PAE) plot of full length DTX3L indicates regions of known domains (RING/CT) and four ‘domains’ in the N-terminus (D1-D4). B) Domain boundaries used in this study and truncations used in interaction mapping (summarised in Table 1). C) Co-immunoprecipitation (Co-IP) assay using over-expressed constructs in HEK293T cells using GFP-TRAP beads with GFPf fusions of 9C and 14C co-expressed with untagged full length (FL) DTX3L or a truncated (D1-4) containing no RING-CT domains. Inputs and bead eluates were blotted for DTX3L (top) and flag (bottom). D) Anti-FLAG Co-IP of GFPf, 9F and 14C co-expressed with HA tagged DTX3L D4 (KLH4-KLH5).

DTX3L had been reported to consist of three domains of undetermined structure N-terminal to the RING and C-terminal domain.^8^, ^11^ It was established that PARP9 interacts with residues 230–540 of DTX3L.^11^ A prediction of DTX3L structure was made using AF2 and the predicted aligned error (PAE) plot was examined (Figure 6A). Instead of three domains, the pre-RING region clearly contained four structural units represented by the distinct blocks along the diagonal. Substantial unstructured linkers were present between domains and all four domains and the RING-CT region were of uncertain orientation with respect to each other. We refer to these pre-RING regions as D1 (approximately residues 1–95), D2 (130–200), D3 (230–365), D4 (370–550). FoldSeek was used to determine closest structural relatives in the protein structure database (PDB), and found with high probability (>0.8) that D1 was similar to the RRM of U1Arnp (1OIA); part of D3 was related to FUSEBP1 (1X4N) and D4 similar to alpha polyC BP (1ZTG) and BICC1 (6GY4) all of which are KH domains (see Figures S8 and S9 for alignment, Table 1). Examination of AF2 models clearly show that D2 had a similar fold to the KHL domains in PARP14 and PARP9 (Figure S8B). The KH pairs of D3 and D4 appear to stack stably against each other and so appear as single structural units in the PAE plot. Again, the KH domains in DTX3L did not contain the canonical GXXG RNA binding motif so we also designated these domains KH-like (KHL). Thus, the pre-RING region of DTX3L is predicted to contain a RRM domain and five KHL domains (Figure 6B).

**Figure 7:**
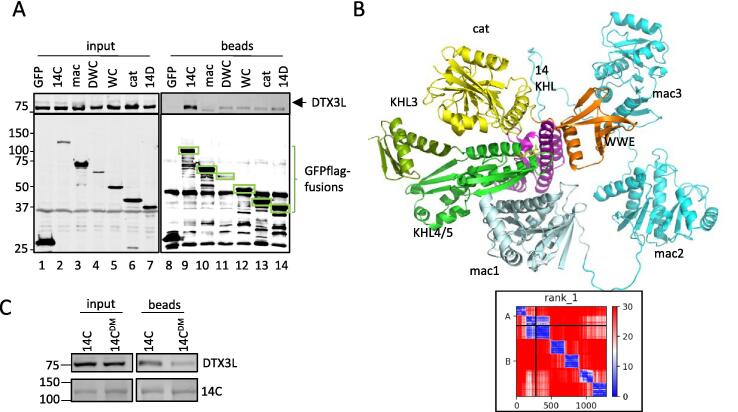
Mapping and prediction of PARP14-DTX3L interaction. A) Anti-flag co-IP of various PARP14 truncation encompassing the C-terminal half co expressed with FL DTX3L. Inputs and beads were analysed by immunoblot using anti-DTX3L (top) and anti-FLAG (bottom). B) Top rank model of DTX3L D3-D4 complexed with PARP14 (14C) created by AF2 ColabFold. with insert of PAE plot of the DTX3L:PARP14 complex pITM = 0.56. C) Anti-FLAG Co-IP of 14C and catalytic double mutant co-expressed with FL DTX3L.

**Figure 8:**
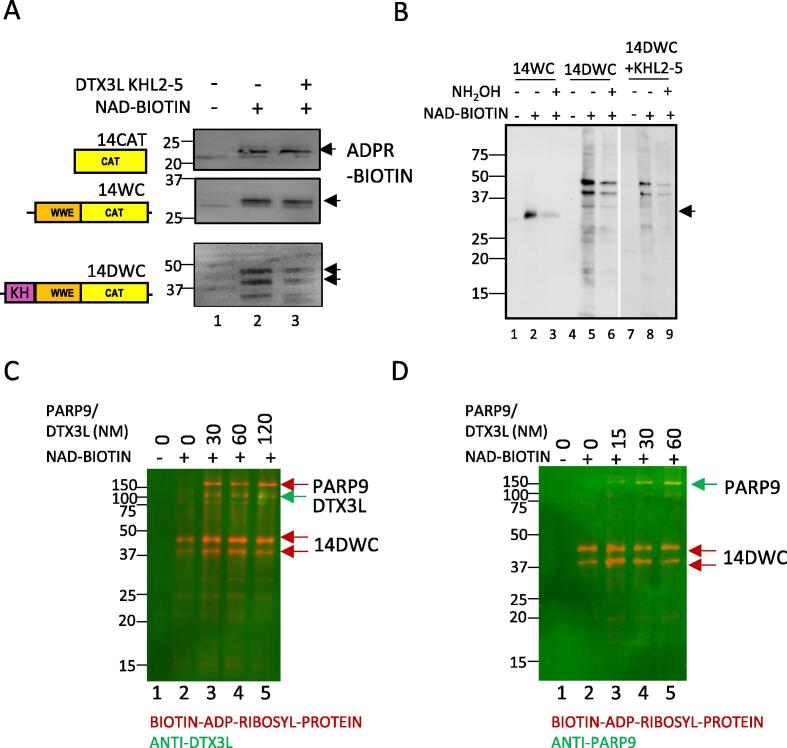
DTX3L interaction influences PARP14 ADP-ribosylation.. A) ADP-ribosylation assay using NAD^+^-biotin and 1 μg PARP14 constructs containing catalytic domain (CAT) or WWE or 14D/KHL domain (14DWC). 1 μg DTX3L D3-D4 was added before reaction was initiated with -NAD+ -biotin. Arrows indicate ADP-ribosylation products. B) 1 M Neutral hydroxylamine was incubated with the reaction products of the ADP-ribosylation assay to identify of modification of acidic residues for each PARP14 construct and in the presence of DTX3L KHL2-5 (expected position indicated by arrow). C) 190 nM PARP14 DWC truncation was included in an ADP-ribosylation assay in the presence of PARP9/DTX3L. Biotinylated ADP-ribosylation products (red) and the DTX3L species are detected with a specific antibody (green) such that ADP-ribosylated DTX3L appears yellow. D) Similarly, the upper band detected in the ADP-ribosylation assay was identified as PARP9 using a specific antibody (green).

An interaction assay was established in HEK293T cells using overexpressed GFP-flag tagged PARP9 and PARP14 constructs and untagged DTX3L constructs. HEK293T do not contain detectable levels of endogenous PARP9, PARP14 or DTX3L (Figure 1A) in this assay, making the cells suitable for mapping interactions. Full length DTX3L and a truncated version lacking the RING-CT domain were able to interact with GFP-flag tagged PARP14 (C-terminus) and PARP9 (Figure 6C, lanes 9–12). Furthermore, a fragment containing predicted domains D1-3 (containing RRM-KHL1-KHL2-KHL3) failed to co-purify with PARP9 and PARP14 (Figure S10C, lanes 9 and 11), and which through elimination, indicates that the interaction site is at D4 (corresponding to KHL4-KHL5). This was confirmed by co-expressing a DTX3L D4 construct which was stabilised by, and copurified with PARP9. The lower affinity interaction was also detected in the PARP14C pull-down (Figure 6D).

Next, the PARP14 domains and truncations (listed in Figure 4A) were tested for interaction with untagged FL DTX3L using a GFP-flag pull-down assay. It was established that the C-terminus of PARP14 (14C) displayed similar levels of interaction with DTX3L as the FL PARP14 protein (Figure S10A). No interaction was detected with the macrodomains alone but removing them also weakened the interaction (compare lane 9 and 11, Figure 7A). No truncation could provide the interaction of the whole C-terminus domain suggesting that several domains coordinate to form the binding site (lanes 11–14, Figure 7A).

The mapping data obtained was supported by an AF2 model of the complex between PARP14 (14C) and DTX3L KHL2-5 obtained by ColabFold (Figure 7B). The top ranked model depicts DTX3L KHL4 sandwiched between macrodomain 1 (mac1) and the catalytic domain and abutting the KHL of PARP14. The pITM value is a measure of confidence in the correctness of the interface between two polypeptides. The top rank pITM was 0.56 and considerably higher than the other models (0.15–0.2, Figure S11A). Notably, only the PAE for top rank model expressed any confidence (blue) *between* residues of the two proteins, more specifically KHL4/5 of DTX3L and the mac1 domain (Figure 4B, inset). The top rank model suggested a proximity between the catalytic domain and the DTX3L-KHL4, albeit with lower confidence than the interaction with mac1. The catalytic mutant of 14C was assessed for binding to DTX3L in the HEK293T co-IP assay. Mutation of two of the catalytic triad residues H1682/Y1714 copurified with reduced amounts of DTX3L supporting the predicted proximity of DTX3L with PARP14 catalytic domain (Figure 7C).

### Interaction of PARP14 and DTX3L effects PARP14 ADP-ribosylation *in vitro*

The proximity of the PARP14 catalytic domain to DTX3L led us to investigate whether DTX3L binding could alter the catalytic activity or chemistry of PARP14 as seen with other PARP family members (PARP9-DTX3L, PARP1-HPF1 and PARP2-HPF1). Bacterially expressed C-terminal fragments of PARP14 including the KHL, WWE and CAT domains and DTX3L KHL2-5 (230–550) were purified and included in an ADP-ribosylation assay (Figure 8A). Expectedly, addition of DTX3L KHL2-5 did not affect the activity of isolated PARP14 catalytical domain (14cat) or WWE-CAT (14WC) proteins because much of the interface was not present. However, the DWC construct which contained the KHL domain, although purified with two species, resulted in reduction in ADP-ribosylation when DTX3L KHL2-5 were added (Figure 8A, lane 3).

Neutral hydroxylamine treatment of PARP14 ADP-ribosylation was used to determine the chemistry of the modification. Neutral hydroxylamine can hydrolyse modifications on acidic residues (Asp, Glu and COOH termini). Indeed, the bulk of the ADP-ribosylation on the PARP14 constructs was removed on 30-minute hydroxylamine treatment (Figure 8B, lanes 3, 6, 9). Furthermore, the addition of DTX3L KHL2-5 did not alter the chemistry of PARP14 auto-ADP-ribosylation modification. Interestingly, very little trans-ADP-ribosylation of the DTX3L fragment was observed at 33 kDa (Figure 8B lanes 8–9, arrow). However, when full length PARP9/DTX3L (purified from overexpression in HEK293T) was added to the reaction, trans-ADP-ribosylation of PARP9 and DTX3L was detected (Figure 8C,D, lanes 3–5). Thus, the data shows that binding of DTX3L to PARP14 suppresses auto-ADP-ribosylation of acidic residues and can promote trans-ADP-ribosylation of full length PARP9/DTX3L; further demonstrating a tertiary complex of PARP14, PARP9-DTX3L can occur using purified components.

### The KHL domain of PARP9 also interacts with a KHL4/5 domains in DTX3L

**Figure 9:**
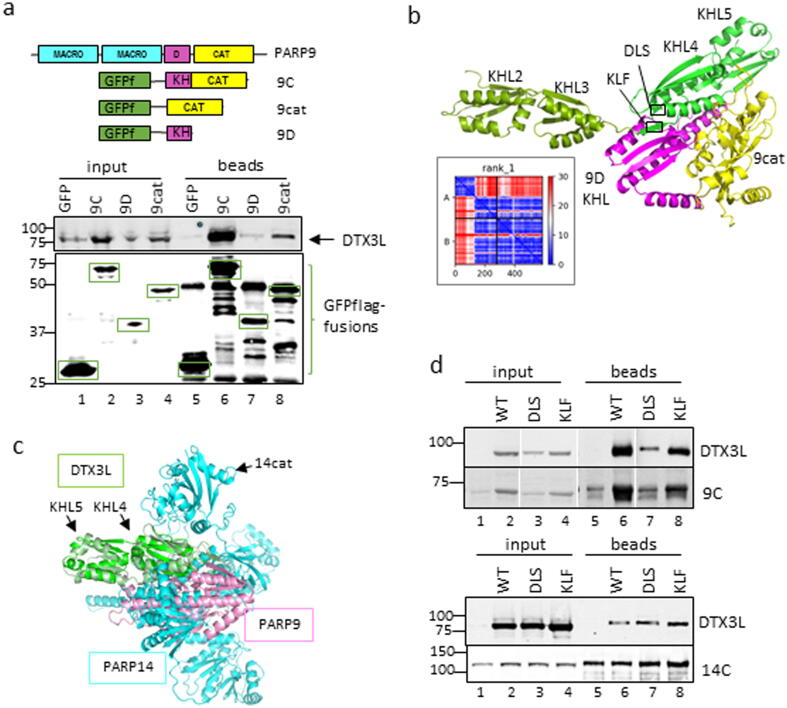
Mapping and prediction of PARP9-DTX3L interaction. A) Depiction of GFP-flag tagged PARP9 were included in an anti-FLAG co-IP of PARP9 truncations co-expressed with DTX3L and probed with anti-DTX3L (top blot) or anti-FLAG (bottom blot). B) AF2 prediction of DTX3L KHL2-5 complexed with PARP9 truncation containing PARP9 KHL and 9cat domains. Insert of PAE plot showing high confidence in arrangements of DTX3L (KHL2-5) and PARP9 interactions pITM = 0.9. C) Superimposed competing PARP9 (pink) and PARP14 (blue) interaction with DTX3L KHL4 (green). D) Three alanine substitutions in DTX3L KHL4 were made in two regions identified in the model for potential contacts with PARP9. FL DTX3L DLS/AAA and KLF/AAA were co-expressed with PARP9 9C (top) or PARP14 14C (bottom) which was pulled down with anti-FLAG beads and blotted for DTX3L and FLAG. Lanes have been rearranged (top) from the same blot to be consistent with anti-FLAG co-IP of PARP14C.

The higher affinity interaction of PARP9-DTX3L does not appear to require macrodomains as shown by similar amounts of DTX3L pulled down by full length PARP9 (9F) and when macrodomains are omitted in 9C truncation (Figure 9A, Figure S10B). The catalytic domain alone (9cat) had a weaker interaction and PARP9 KHL domain showed no independent interaction with DTX3L and both domains were required for strong interactions (compare lane 6 and 8, Figure 9A). The AF2 models of PARP9 (9C) and DTX3L (KHL2-5) complex predicted an interaction with DTX3L KHL4 and showed that the PARP9 KHL bridged the catalytic domain and DTX3L at several places (Figure 9B). The PAE plot showed high confidence between the KHL4 and KHL5 domains of DTX3L and both domains of the 9C construct (Figure 9B, inset). Furthermore, the 5 top ranked models were consistent in their orientation and possessed high pITM values (0.88–0.9) and so represented a very confident prediction of the interaction (Figure 9B, Figure S12).

**Figure 10:**
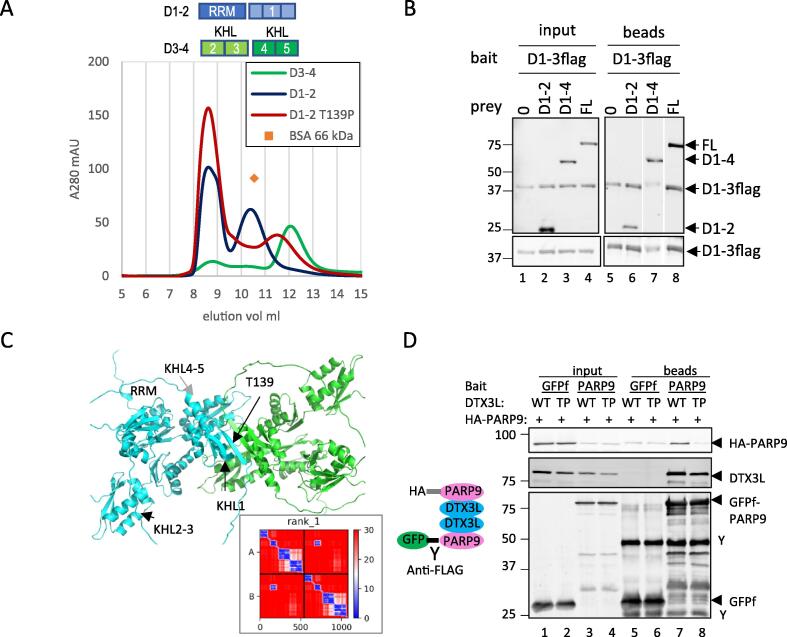
Homodimerisation of DTX3L through KHL1. A) Size exclusion chromatography of purified domains on Superdex-75 column. The domains are represented in the cartoon. B) Anti-FLAG co-IP of flag-tagged DTX3L D1-3 with untagged FL and truncated DTX3L. Inputs and beads were blotted with anti-DTX3L (top) and anti-FLAG (bottom). Lane 7 was rearranged to follow a logical order. C) Top-ranking AF2 structure and PAE plot of DTX3L homodimer indicating confidence in KHL1 interaction. Dimeric KHL1 interaction occurs through augmentation of β-sheets with conserved T139 lying central in the β1-strand. D) FLAG co-IP of co-expressed GFPf-PARP9 and HA-PARP9 with DTX3L WT or T139P. HA-PARP9 co-purifies when the homodimerisation interface of DTX3L is intact.

Interaction results and AF2 predictions indicated that both PARP9 and PARP14 interacted with DTX3L KHL4, and might share a common interface. Superimposing the interactions onto KHL4/5 show a common interface with the exception of the catalytic domain that interacted with the opposing side of DTX3L KHL4 (Figure 9C). Certain residues in DTX3L-KHL4 were identified that appeared to form contacts with PARP9-KHL using the highest confidence DTX3L-PARP9 model (Figure 9C). Two three amino-acid regions of possible contacts (KLF 367–369 and DLS 428–430) were selected from the model which were also conserved between species. Mutations to alanine were made for expression in the interaction assay. A reduction of PARP9 and DTX3L-DLS/AAA expression suggested a disruption of interaction and resulted in a decrease in DTX3L co-precipitated (Figure 9D, top, lanes 3 and 7). DTX3L KLF/AAA mutation did not affect interaction or expression levels to the same extent (Figure 9D, lanes 4 and 8). In contrast, 14C interacted normally with the DTX3L-DLS mutant, suggesting differences in the manner of interaction between DTX3L-PARP9 and DTX3L-PARP14 (Figure 9D bottom lane 7). In summary, the KHL domains in PARP9 and PARP14 are each predicted to interact with the KHL4 domain in DTX3L but appear to involve different contacts. (Figure S13).

### Dimeric interactions take place in D1-D2 domains of DTX3L through KHL1 domain

A tertiary complex of PARP9-DTX3L and PARP14 has been reported,^19^ leading us to hypothesise that DTX3L might exist in a multimeric state to allow both PARPs to interact with DTX3L KHL4. Bacterially expressed constructs D1-2 (RRM-KHL1) and D3-4 (KLH2-5), were affinity purified through a His-tag fusion followed by size-exclusion chromatography which would give an indication of size and oligomeric state. D1-2 and D3-4 each have an estimated molecular mass of ∼33 kDa from sequence and SDS-PAGE. However, D1-2 eluted at approximately the same time as monomeric BSA (66 kDa) whereas D3-4 eluted at later times with smaller proteins (Figure 10A). This indicated that D1-2 was dimeric and D3-4 was monomeric. A co-IP assay was performed using flag-tagged D1-3 (RRM-KHL1-3) and various DTX3L truncations. All DTX3L truncations co-purified with D1-3 including D1-2 also mapping the dimeric interface between residues 1–230 (RRM-KHL1) (Figure 10B).

Once more AF2 was used to predict an interaction and determine how a dimer of DTX3L (minus the RING-CT domains) might be arranged. The PAE plot suggested there was low confidence in inter-domain and inter-chain interactions with the striking exception of a region corresponding to KHL1 domain (Figure 10C, inset). The model showed an interface composed of antiparallel β -sheets (Figure 10C). To disrupt this structure a conserved threonine T139 (numbering from Uniprot Q8TDB6-1) was mutated to a proline in the D1-2 expression construct. On purification, this mutation changed the elution profile of soluble domains to a position between monomeric and dimeric positions indicating that the mutation destabilised the dimer interaction (Figure 10A). The D1-2 protein is not particularly soluble and tends toward aggregation, the mutation more so: this might be explained by large unstructured regions as depicted in the AF2 model (Figure 10C). The pITM values are low in the top 5 D1-4 models (0.25) despite the pLDDT values being high across the augmented β -sheet (Figure S14). Conceivably, the relatively modest size of the predicted interface may depress the confidence in the interaction that can be achieved. Homodimerisation of DTX3L through KHL1 was supported in the interaction assay using full length PARP9 and DTX3L. Co-expression of GFPf and HA tagged PARP9 with DTX3L resulted in both PARP9 versions being eluted in the anti-FLAG co-IP (Figure 10D, lane 7). Mutation of DTX3L T139 to proline (TP) greatly reduced HA-PARP9 being eluted despite similar levels of GFPf-PARP9 and DTX3L (WT and mutant) being pulled down by the anti-FLAG beads (Figure 10D, lane 7 and 8). Structurally, PARP9-DTX3L forms a dimer through the KHL1 domain of DTX3L.

## Discussion

This study aimed to characterise the interactions and structural requirements of the complex of PARP9/DTX3L and PARP14 which is implicated in survival of several cancer cell types. Using structure-prediction tools we identified regions of unknown structure in the C-terminus of PARP14 and PARP9 as KH-like domains. Typically type I eukaryotic KH domains are RNA/DNA binding but we showed they can also mediate protein–protein interactions. We also identified five KH domains and a RRM in DTX3L. Both PARP9 KHL and PARP14 KHL contributed to interactions with DTX3L KHL4 but differed in their stable contacts. KHL1 in DTX3L provides a homodimerization interface which allows a dimeric complex of PARP9-DTX3L and a tertiary complex of PARP9-DTX3L-PARP14 to form.^19^ Characterising the interactions of KHL domains is important to the understanding of how PARP9-DTX3L-PARP14 stabilise their protein levels and which domains are important for promoting cancer cell survival.

The regulation of PARP14 protein levels by PARP9/DTX3L becomes apparent when these proteins are depleted by siRNA. PARP9/DTX3L knockdown does not reduce PARP14 mRNA levels so regulation occurs at translation or post-translation stages. PARP14 protein is rapidly removed (half-life < 3 h) in the absence of PARP9/DTX3L in an unknown mechanism that is independent of proteasomal or lysosomal degradation pathways.

Depleting PARP9, DTX3L and PARP14 levels reduced cell survival and proliferation, with an apparent increase in apoptosis in various HNSCC cell lines and HeLa cells. A similar effect on PARP14 expression and survival was seen in PARP9, DTX3L and PARP14 depleted HeLa and BxPC3 PAAC cell lines and it is possible that overexpression of PARP9 and PARP14 is a common survival pathway in a variety of cancer cells, in addition to metastatic prostate cancer cell lines previously reported.^19^ We note, the PARP14-mediated survival was particularly evident in FaDu, Detroit 562, BXPC3 (this study) and PC3m (previously reported) where PARP14 levels were comparatively high and TP53 is mutated. However, TP53 status was not associated with high PARP14 levels. PARP9/PARP14 double knockdown did not produce an additive reduction suggesting the effects on cell survival were on the same pathway. Since PARP9 levels effect PARP14 expression, it was reasonable to assume that the colony formation phenotype this working through PARP14 action. Interestingly, the catalytic inhibitor RBN012759 did not alter survival and a catalytic mutation of PARP14 was still able to partially rescue this phenotype in PARP14–depleted HeLa cells. It had been suggested using the PARP inhibitors DPQ or TIQ-A, that PARP14 activity is required for metastatic prostate cancer cell line survival, but these are not particularly selective and could be inhibiting other PARP family members.^27^, ^28^

Eukaryotic type I KH domain (KH) are present in several RNA and DNA binding proteins and possess a GXXG loop and local basic charge necessary for nucleic acid binding, features not present in the C-terminal PARP or N-terminal DTX3L structures. Recently, AF2 was also used to identify KH domains in PARP14 in the N-terminal half of PARP14 which did have RNA binding properties.^29^ Although we did not rule out nucleic acid binding in the KHL in the C-terminus chemically it seemed unlikely. None of KHL domains in DTX3L have the canonical GXXG motif but the presence of RRM at the N-terminus would indicate that RNA/DNA binding might be possible and worth exploring perhaps in the context of viral RNA binding or DNA damage. It is common to find KH domains in tandem which acts to extend nucleic acid recognition from 4 bases per domain. Interestingly the single KH domains in PARP9, PARP14 and DTX3L (KHL1) were involved in protein–protein interactions and their propensity to form pairs might be the basis of protein–protein interaction.^30^

The Deltex family of E3 Ub ligases exist as homo- or hetero- dimers and DTX3L can form homodimers or heterodimers with DTX1.^31^, ^32^ We confirmed DTX3L homodimerisation and show it also occurs in the presence of PARP9. The dimerization site was mapped to residues 1–230 and AF2 predicted the interface formed an augmented β -sheet, as observed in some KH domain crystal packing.^30^ Disruption of the β 1 strand by T139P mutation abolished dimerisation. Biophysical analysis by a recent study also identified D2 providing the dimer interface.^33^ The oligomeric state of PARP9/DTX3L was also explored in the same study that also used intra- and inter- strand crosslinking with BS^3^ to identify contacts. The crosslinker was unable to identify D2-D2 interactions, possibly due to the lack of proximal lysines in the β -strands, but they identified K363 and K401 (in DTX3L KHL4) crosslinking with PARP9 K557 residues (KHL-C) agreeing with the findings we made in this study.^33^ We identified DLS (428–430) residues in DTX3L KHL4 that effected interactions with PARP9, but not PARP14.

We show that DTX3L is proximal to the PARP14 catalytic domain; verified by interaction assays using PARP14 catalytic mutations, which reduced DTX3L binding. Furthermore, DTX3L binding could suppress PARP14 auto-ADP-ribosylation activity *in vitro* and promote trans-ADP-ribosylation of full length PARP9 and DTX3L, a clear sign of tertiary interaction. The ADP-ribosyl- acceptor sites on DTX3L are likely to occur at N- or C- terminal domains because KHL2-5 could block auto-ADP-ribosylation without greatly stimulating trans-ADP-ribosylation. Unlike the PARP1-HPF1 interaction, there was no evidence that DTX3L interaction for altered the chemistry of the acidic acceptor sites.^34^ Since cancer cell survival was not dependent on PARP14 catalytic activity we postulate that PARP9/DTX3L regulates PARP14 expression and activity to promote proliferation and anti-apoptosis.

In summary, we have provided insight into the structure and interactions between two macro-PARP family members and DTX3L that have several pro-tumour functions. While specific PARP14 inhibitors were unable to suppress survival mechanisms it might be achieved if PARP9/DTX3L was targeted. However, no specific inhibitors to this complex have yet been developed.

## Materials and Methods

### Cell culture

HeLa (RRID:CVCL_0030, ATCC) and HNSCC cell lines, UMSCC74A (RRID:CVCL_7779), UMSCC6 (RRID:CVCL_7773), UMSCC17A (RRID:CVCL_7724), UMSCC11B (RRID:CVCL_7716), SCC154 (RRID:CVCL_2230), SCC90 (RRID:CVCL_1899); kind gifts from Thomas Carey, University of Michigan and A253 (RRID:CVCL_1060, ATCC), were cultured in high glucose DMEM 10% FBS (Gibco), 1 mM Glutamine or Glutamax, non-essential amino acids and penicillin/streptomycin (Sigma). FaDu (RRID:CVCL_1218, ATCC) and Detroit 562 (RRID:CVCL_1171, ATCC) were cultured in MEM plus the same additives. PAAC cell lines MIA PaCa2 (RRID:CVCL_0428), PANC-1 (RRID:CVCL_0480) and BxPC-3 (RRID:CVCL_0186) were gifts from Dr Pedro Perez-Mancera (University of Liverpool, UK), Prostate adenocarcinoma cell lines (PRAD) 22RV1 (RRID:CVCL_1045) and PC-3 M (RRID:CVCL_9555) were a gift from Prof Ke (University of Liverpool, UK) all grown in DMEM media as above. HEK293T (RRID:CVCL_0063) were cultured in DMEM:F12 media supplemented with 10% FBS, Pen/Strep and glutamine.

The cell lines have been authenticated by STR profiling (Northgene, Deeside, UK). The loci tested include the 17 markers contained within ICLAC recommended databases in compliance with ASN-0002–2022. The allelic data from samples was compared against cell line database. Cell lines were mycoplasma tested regularly using e-Myco PCR kit (Chembio Ltd, UK).

### Inhibitors

The proteasome inhibitor, MG132(#A2585) was purchased from Stratech Scientific and used at a concentration of 10 μM. The autophagy inhibitor, Chloroquine (#14774) was obtained from Cell Signalling Technology (CST) and used at a concentration of 10 μM. Cycloheximide (#C81040), sourced from Research Products International, was used at 100 μM. The PARP14 catalytic inhibitor (RBN012759) and negative control (RBN250039) were provided by Ribon therapeutics (Cambridge, MA, USA) and used at 0.1 μM (following optimization between 10 nM and 10 μM).^26^

### Immunoblotting

Cell pellets were harvested by scraping into ice-cold PBS and total soluble protein extracts obtained.^35^ Equal amount of total protein (25–40 μg, estimated by Bradford reagent, Bio-rad) were separated by 8% or 10% Tris-Glycine SDS-PAGE and transferred to 0.45 μm PVDF (low fluorescence, Millipore). The membrane was blocked using Incept buffer (LI-COR Biosciences) and antibodies were prepared in 50% Incept buffer in PBS/0.01% Tween 20. Antibodies used: PARP9 (Figure 1A, S1A, Abcam, ab53796 1:500; all other immunoblots, Proteintech, 17535-1-AP, 1:500); DTX3L (CST, #14795, 1:1,000); PARP14 (Abcam ab229756, 1:500); actin (Merck, A5441, 1:20,000), PARP1 (Proteintech, 66520-1-Ig, 1:1,000), pJNK1 (CST 81E11 1:1,000) JNK2 (CST, 56G8, 1:1,000) p62/SQSTM1 (CST #5114, 1:1000) and detected using IR-800 (LI-COR, 926-32210 or 926-32211, 1:10,000) or Alexafluor-680 (Invitrogen, A21057 or A21076 1:10,000) conjugated goat secondary antibodies. Fluorescent blots were scanned with Odyssey (LI-COR) and bands quantified using ImageJ software.

### qPCR analysis

Total RNA was isolated using the Qiagen RNeasy RNA extraction kit (Qiagen, UK) following the manufacturer's instructions. Subsequently, 1 µg of the total RNA was subjected to reverse transcription using the GoScript kit (Promega, US) as per the supplier's protocol. The design of primers and probes for PARP9, PARP14, and DTX3L was facilitated by the PrimerQuest design tool in IDT Sci Tools, and the synthesis of these primers and probes was performed by IDT. The reaction was performed in a 7500 Fast RT-PCR system (Life Technologies/Applied Biosystems) under the following thermal conditions: initial denaturation (95 °C, 2 min), denaturation 40 cycles, 95 °C, 5 s), annealing (56 °C, 15 s) and extension (60 °C, 45 s). The cycle threshold (CT) values of the target genes were identified, and mRNA levels were calculated as a ratio of normalised ACTB level according to the 2^−ΔΔCT^ method. See Table S1 for sequences.

### Plasmid vectors

PARP9 and PARP14 fragments with overlapping vector regions were assembled in to pEGFP-3flag vector (Addgene plasmid # 46957^36^ using HiFi NEB builder (NEB). GFP-PARP14 were provided by Michael Cohen (Oregon Health and Science University, USA)^37^ pKH3-PARP9-HA and pKH3-DTX3L were provided by Bryce Paschal (University of Virginia, USA).^7^ Truncations and mutations were created using primers listed in Table S2.

### Transfections

Three individual siRNAs were designed and purchased from IDT (IDTdna.com, sequences listed in Table S3). 150,000 cells seeded in 35-mm dishes were transfected with 2 μl lipofectamine RNAiMAX (Invitrogen) and 1 nM siRNA in OptiMEM (Gibco) 24 hours later using manufacturer’s instructions. Cells were used 48 h later or otherwise stated. For complementation, recombinant plasmid expression was performed 24 h following knock-down transfection using 2 μl lipofectamine 2000 (Invitrogen) and 1 μg DNA. The cells were used in clonogenic assays 24 h later. GFP-fusion expression analysis was performed in HeLa cells using the plasmid transfection conditions above then viewed on an EVOS microscope with GFP filter at 20X magnification.

### Colony formation assay

Clonogenic survival assays were performed as previously described.^38^, ^39^ In brief, siRNA experiments, 1.5 × 10^5^ cells were seeded in 35-mm dishes and allowed to attach overnight in 5% CO_2_, 37 °C humidified incubator. Cells were then transfected and incubated for 48 h after which they were harvested and a defined number of cells was seeded in triplicates in 6-well plates. For clonogenics using PARP14 catalytic inhibitor, cells were pre-seeded into 6-well plates and allowed to attach for at least 6 h after which 0.1 μM RBN250039 (control compound) and RBN012759 or DMSO control were added.^26^ Colonies were allowed to grow for 7–12 days, following which they were fixed and stained with 6% glutaraldehyde and 0.5% crystal violet for 1 h. The plates were subsequently washed and left to air dry overnight, and colonies were counted using the GelCount colony analyser (Oxford Optronics, Oxford, UK). Relative colony formation was expressed as colonies per treatment level versus colonies that appeared in the untreated control. The data obtained for this analysis was derived from a minimum of three individual biological replicates.

### Proliferation assay

CyQUANT cell proliferation assay kit (Invitrogen, USA) was used to assess cell proliferation following PARP9, DTX3L and PARP14 depletion, following manufacture’s protocol. Fluorescence was then measured at 520 nm following excitation at 480 nm using a Spark multimode Tecan microplate reader.

### Structure predictions and web-based tools

Initial analysis of predicted structure was obtained from the AlphaFold Protein Structure Database (alphafold.ebi.ac.uk).^40^, ^41^ Interaction studies were then performed using ColabFold.^42^ Prediction of secondary structure was performed at PSIPRED (bioinf.cs.ucl.ac.uk) and domain identification by FoldSeek (search.foldseek.com).^43^ Sequence alignment was performed using T-coffee (ebi.ac.uk/Tools/msa/tcoffee/).^44^ Structures were viewed, aligned and annotated with PyMOL 2.1.1 open source software.

### GFP/FLAG co-immunoprecipitation Interaction assays

Co-IPs were performed using 20 μl GFP-trap nanobodies (Chromotek) or M2-agarose anti-FLAG resin (Sigma) using interacting proteins transiently expressed in HEK293T.^45^ Modifications included 1 × 10^6^ cells were seeded in 10-cm dish and the following day 24 μg polyethylenimine and 8 μg total DNA were mixed in 0.8 ml Hybridoma SFM and added to the dish containing 7.2 ml Hybridoma SFM, 1% FBS. Cells were washed and harvested 48 h after transfection.

### Protein expression and purification

PCR constructs encoding PARP14 and DTX3L truncations were inserted into pET28a vectors (C-terminal His-tag) using HiFi NEB builder (Table S2). Sequence verified vectors were transformed into BL21 DE3 pLysS (NEB) cells and grown on Luria Broth Agar (Fisher) with 50 μg/ml kanamycin. 50 ml cultures were grown overnight and used to inoculate 500 ml LB/kan at 37 °C. When OD600 ∼0.6 cultures were cooled on ice, induced with 0.1 mM Isopropyl β-d-1-thiogalactopyranoside and cultured at 16 °C overnight. Harvested cells were frozen before lysis in 25 mM Tris-HCl pH 7.8, 0.5 M NaCl, 10 mM imidazole pH 8, 5% glycerol, 1.4 mM β-mercaptoethanol, 100 μM phenylmethylsulfonyl fluoride. Cell suspension was sonicated 3 × 30 s on ice and cleared by centrifugation (10,000*g*, 10 min. 4 °C). Supernatant was loaded on 1 ml HisTrap FF columns (GE Healthcare), washed in buffer containing 25 mM imidazole and eluted in 250 mM imidazole. Fractions were pooled and concentrated then injected onto Superdex 75 column (GE Healthcare) equilibrated in gel filtration buffer (20 mM Tris-HCl pH7.5, 150 mM NaCl, 5% glycerol, 1 mM dithiothreitol) and collecting and snap freezing the main fraction (not aggregate). Protein purity was analysed by 15% SDS PAGE.

GFPf-PARP9/DTX3L coexpressions in HEK293T were performed in four 10-cm dishes as in section 2.10.^45^ 1 ml supernatant was applied to 100 μl M2 anti-FLAG agarose, incubated for 1 h, 4 °C then washed three times in IP buffer and twice in gel filtration buffer (no DTT). Protein was eluted by three 5-min incubations with 0.5 ml 0.2 M glycine-HCl pH2.5, neutralised by 0.2 M Tris-HCl pH7.5, concentrated and buffer exchanged using a 0.5 ml Amicon Ultracel 10 K centrifugal filters (Millipore) with three dilutions of gel filtration buffer.

### ADP-ribosylation assay

Reactions containing 1 μg PARP14 and 12.5 μM NAD-biotin (Biotechne) and optionally 1 μg DTX3L D3-4 in 1X NEB buffer 1 were incubated at room temperature for 30 min.^46^ 1 M hydroxylamine treatments were performed for a further 30 min by adding 1 vol. 2 M solution, pH 7.0. Reactions were stopped with SDS-PAGE loading buffer (not heated) and separated on 15% Tris-Glycine SDS-PAGE gel. Products were transferred to PVDF, blocked with 5% bovine serum albumin (BSA) TBS (25 mM Tris-HCl pH7.5, 150 mM NaCl) then probed with 1:14,000 streptavidin-AlexaFluor 680 (Invitrogen) in 1% BSA TBS 0.01% Tween 20 (TBS-T) for 1 h, washed three times in TBS-T plus 0.04% SDS before scanning on an Odyssey instrument (Li-COR).

### CRediT authorship contribution statement

**Hadil Saleh:** Formal analysis, Investigation, Methodology, Writing – original draft, Visualization. **Triantafillou Liloglou:** Methodology, Resources, Software, Supervision, Writing – review & editing. **Daniel J. Rigden:** Formal analysis, Methodology, Supervision, Writing – review & editing. **Jason L. Parsons:** Project administration, Resources, Supervision, Writing – review & editing. **Gabrielle J. Grundy:** Conceptualization, Formal analysis, Funding acquisition, Investigation, Methodology, Project administration, Resources, Supervision, Visualization, Writing – original draft.

## Declaration of competing interest

The authors declare that they have no known competing financial interests or personal relationships that could have appeared to influence the work reported in this paper.

## Data Availability

Data will be made available on request.
