# Supplementary material for: KH-like Domains in PARP9/DTX3L and PARP14 Coordinate Protein–Protein Interactions to Promote Cancer Cell Survival
Source: J Mol Biol. 2024 Feb 15;436(4):168434. doi: 10.1016/j.jmb.2023.168434 (PMC11080071; doi:10.1016/j.jmb.2023.168434)
Supplement: Supplementary data 1 [file mmc1.docx]

## Supplementary Information

## Supplementary Methods

### Cell cycle analysis

HeLa and FaDu cells, seeded at 4 X 10^5^ cells per 6 cm dish, were transfected with siRNAs targeting PARP9, PARP14, or DTX3L. Following a 48 h incubation period, cells were harvested by trypsinisation, and pelleted by centrifugation at 300 x *g* for 5 min at 4°C. After washing with cold PBS, the pellets were fixed with ice-cold 70% ethanol and maintained on ice for a minimum of 30 min before staining. The fixed cells were centrifuged at 300 x *g* for 5 min at 4°C, washed twice with cold PBS and resuspended in 200 mL of cold PBS. To facilitate cell cycle analysis, fixed cells were stained with 7-Aminoactinomycin D at a final concentration of 25 µg/ml for 30 min in the dark. The stained cells were subjected to cell cycle analysis using Attune Nxt Flow cytometer (Invitrogen) and population of cells in each phase was determined using its associated software.

### Cell fractionation

Biochemical fractionation to produce soluble and chromatin-bound proteins was performed as described previously [1]. Briefly, cell pellets were resuspended in two packed cell volumes (PCVs) of buffer I (20 mM Tris–HCl pH 7.8, 2.5 mM MgCl_2_, 0.5% (v/v) IGEPAL CA-630, 100 µM PMSF, 1 mM N-ethylmaleimide (NEM), and 1 µg/mL of the protease inhibitors, incubated for 10 min on ice and centrifuged at 10,000 rpm for 2 min at 4°C. The resulting the supernatant containing soluble proteins (C) was collected. The nuclear pellet was similarly extracted with two PCVs of buffer II (20 mM NaPO_4_, pH 8.0, 0.5 M NaCl, 1 mM EDTA, 0.75% (v/v) Triton X-100, 10% (v/v) glycerol, 100 µM PMSF, 1 mM NEM, and 1 µg/mL protease inhibitor) and incubated on ice for 10 min. After centrifugation, the supernatant containing nuclear proteins (N) was collected. Immunoblotting analysis using 40 µg of protein from the C fraction and the N fraction were used, and proteins were visualized using the Odyssey image analysis system (Li-cor Biosciences).

### Table S1: List of Gene specific primers and probes used in qPCR experiments.

| **Gene** | **Forward 5’-3’** | **Reverse 5’-3’** | **Probe 5’-3’** |
| --- | --- | --- | --- |
| PARP9 | 5GTGGTATGCAGAGTTGGCTT | CTCTGCCAGGTTTTTGAGG | AGAATGTACTCGACACCTTGCGATCC |
| PARP14 | CAGAAGGGCTAATGGAAATC | TGAATCAGGGCTAGAAACAGT | AATTCCGAAGGCTAAAGATACACAAGGT |
| DTX3L | AATTCCGAAGGCTAAAGATACACAAGGT | TCGCTGTATTCCAGGGTATCT | ΑACAGAAGAACACCCAAACCCAGGA |
| ACTB | CCAGCACAATGAAGATCAAGATCA | CATACTCCTGCTTGCTGATCCA | CTCCTCCTGAGCGCAAGTACTCCGTG |

### Table S2: PCR primers used to create truncated and mutant constructs

| **Construct** | **Primers (5’-3’)** | **Template** | **Destination** | **Final** |
| --- | --- | --- | --- | --- |
| 14N | P14FLF:GATGACGACGATAAGCTCGAGATGGCTGTGCCCGGC  P14NR:GTTATCTAGATCCGGTGGATCCTTAGCCTCCCTCCTTCATTACTTCATTCTCCTG | pEGFP-PARP14 | pEGFP-3flag (Xho/BamHI) | pGFP-flag-P14N |
| 14C | 14mac1F:GATGACGACGATAAGCTCGAGGCTGGGCAGAAGTGCTTCTCTCG  14FLR:GTTATCTAGATCCGGTGGATCCTTATTTTCTAAACGTAATAAGGTACTC | pEGFP-PARP14 | pEGFP-3flag (Xho/BamHI) | pGFP-flag-P14C |
| 14mac | 14mac1F:GATGACGACGATAAGCTCGAGGCTGGGCAGAAGTGCTTCTCTCG  14mac3R:GTTATCTAGATCCGGTGGATCCTTACTGAGTCCCTTCTCTTTTCTTCATGTTG | pEGFP-PARP14 | pEGFP-3flag (Xho/BamHI) | pGFP-flag-P14-MAC |
| 14macD | 14mac1F:GATGACGACGATAAGCTCGAGGCTGGGCAGAAGTGCTTCTCTCG  CA14R:GTTATCTAGATCCGGTGGATCCCTATTCTTTGGCCAATCGAACTCTCTTGATC | pEGFP-PARP14 | pEGFP-3flag (Xho/BamHI) | pGFP-flag-P14-MACD |
| 14DWC | CA14F:GATGACGACGATAAGCTCGAGCAGCTTTCTTCCCAACAGTCTGTGATG  14FLR:GTTATCTAGATCCGGTGGATCCTTATTTTCTAAACGTAATAAGGTACTC | pEGFP-PARP14 | pEGFP-3flag (Xho/BamHI) | pGFP-flag-P14-DWC |
| 14WC | 14WCF:GATGACGACGATAAGCTCGAGCAGGAATCCCGGGCAGATTGTATCAG  14FLR:GTTATCTAGATCCGGTGGATCCTTATTTTCTAAACGTAATAAGGTACTC | pEGFP-PARP14 | pEGFP-3flag (Xho/BamHI) | pGFP-flag-P14WC |
| 14cat | 14catF:GATGACGACGATAAGCTCGAGAAAGTTGACATCCCTGCACACTGGAG  14FLR:GTTATCTAGATCCGGTGGATCCTTATTTTCTAAACGTAATAAGGTACTC | pEGFP-PARP14 | pEGFP-3flag (Xho/BamHI) | pGFP-flag-P14CAT |
| 14D | CA14F:GATGACGACGATAAGCTCGAGCAGCTTTCTTCCCAACAGTCTGTGATG  CA14R:GTTATCTAGATCCGGTGGATCCCTATTCTTTGGCCAATCGAACTCTCTTGATC | pEGFP-PARP14 | pEGFP-3flag (Xho/BamHI) | pGFP-flag-P14-ca |
| 14Cmut | PARP14H1682AF:GAATGAGAAGCAACTCTTCGCTGGGACAGATGCCGGCTCCG  PARP14H1682AR:CGGAGCCGGCATCTGTCCCAGCGAAGAGTTGCTTCTCATTC | pGFP-flag-P14C | - | pGFP-flag-P14C-HA |
| 14C-DM | PARP14Y1714AF:CATATGGAAAGGGAACCGCTTTTGCTGTCAATGCCAATTATTC  PARP14Y1714AR:GAATAATTGGCATTGACAGCAAAAGCGGTTCCCTTTCCATATG | pGFP-flag-P14C-HA | - | pGFP-flag-P14C-DM |
| DWC-DM | CA14F:GATGACGACGATAAGCTCGAGCAGCTTTCTTCCCAACAGTCTGTGATG  14FLR:GTTATCTAGATCCGGTGGATCCTTATTTTCTAAACGTAATAAGGTACTC | pGFP-flag-P14D-DM | pEGFP-3flag (Xho/BamHI) | pGFP-flag-P14-DWC-DM |
| 9F | 9FLF:GATGACGACGATAAGCTCGAGATGGACTTTTCCATGGTGGCCGGAG  P9R:GTTATCTAGATCCGGTGGATCCTTAATCAACAGGGCTGCCACTTGCGAATC | pKH3-PARP9-HA | pEGFP-3flag (Xho/BamHI) | pGFPf-PARP9 |
| 9N | 9FLF:GATGACGACGATAAGCTCGAGATGGACTTTTCCATGGTGGCCGGAG  9Mac2R:GTTATCTAGATCCGGTGGATCCTTAACTGTAATTGTTCAAACTCAGCATCTTG | pKH3-PARP9-HA | pEGFP-3flag (Xho/BamHI) | pGFPf-P9N |
| 9C | 9CAF:GATGACGACGATAAGCTCGAGCAGCTTTCTTCCCAACAGTCTGTGATG  P9R:GTTATCTAGATCCGGTGGATCCTTAATCAACAGGGCTGCCACTTGCGAATC | pKH3-PARP9-HA | pEGFP-3flag (Xho/BamHI) | pGFPf-P9C |
| 9cat | 9catF:GATGACGACGATAAGCTCGAGATTCAGCAACAAAAAACCCAAGACGAAATG  P9R:GTTATCTAGATCCGGTGGATCCTTAATCAACAGGGCTGCCACTTGCGAATC | pKH3-PARP9-HA | pEGFP-3flag (Xho/BamHI) | pGFPf-P9cat |
| 9D | 9CASF:GATGACGACGATAAGCTCGAGGGGCTTGAAGCTAGATCTCCTGCCATC  9CASR:GTTATCTAGATCCGGTGGATCCTTACTGTCCTAACGAGCGCCAAAGGCCTC | pKH3-PARP9-HA | pEGFP-3flag (Xho/BamHI) | pGFPf-P9D |
| D1-4 | DTX3L_DCF:CTCCGCCACTCAAGGGCTAAGTGAGTTCTGAGGCCTC  DTX3LDCR:GAGGCCTCAGAACTCACTTAGCCCTTGAGTGGCGGAG | pKH3-DTX3L | - | pKH3-DTX3LDC |
| D1-3 | DTX3L_365XF:CTGAAGCTTTTGTCAAGATATAGGTGAAACTATTTGCTGCC  DTX3L365XR:GGCAGCAAATAGTTTCACCTATATCTTGACAAAAGCTTCAG | pKH3-DTX3L | - | pKH3-DTX3L-365 |
| fD1-3 | CMV_DTX3LF:CGCTCTAGCCCGGGCGGATCCATGGCCTCCCACCTGCGCCCG  CMV_DTX3LR:CTTATCGTCGTCATCCTTGTAATCCTCGAGTATCTTGACAAAAGCTTCAGAGATTTTTTGTTTGGC | pKH3-DTX3L | pCMV6  BamHI/XhoI | pCMV-Flag-DTX3L D1-3 |
| D1-2 | DTX3L230XF:CCAGAAACCAAGGCAGAATAATAAAGCAACTATTTTGAAG  DTX3L230XR:CTTCAAAATAGTTGCTTTATTATTCTGCCTTGGTTTCTG | pKH3-DTX3L | - | pKH3-DTX3L-230 |
| D4 | D4HAF:CAACTGCACCTCGGTTCTAAGCTTATGGTGAAACTATTTGCTGCCAATTACATGATG  DAHAR:CTGGAACATCGTATGGGTACATTCTAGATCCTTTTAGAACATACTGCTTCGCCTTTGC | pET28a-DTX3L  D3-4 | pKH3  HindIII/XbaI | pKH3-D4-HA |
| DTX3L-T138P | DTX3L_T139PF:GATTCCTGTCTCCAAAAGATCTTTCTTACTGTACCCGCTGACCTGAACTGTAACCTGTTC  DTX3L_T139PR:GAACAGGTTACAGTTCAGGTCAGCGGGTACAGTAAGAAAGATCTTTTGGAGACAGGAATC | pKH3-DTX3L | - | pKH3-DTX3L-T138P |
| DTX3L-KLF/AAA | DX_KLFF:GCTTTTGTCAAGATACCTGTGGCAGCAGCTGCTGCCAATTACATGATGAATG  DX_KLFR:CATTCATCATGTAATTGGCAGCAGCTGCTGCCACAGGTATCTTGACAAAAGC | pKH3-DTX3L | - | pKH3-DTX3L-KLF/AAA |
| DTX3L-  DLS/AAA | DX_DLSF:GAATCCAAGGACAGGCAGGTAGCTGCAGCTGTGCATGCTTATGCAAGTTTC  DX_DLSR:GAAACTTGCATAAGCATGCACAGCTGCAGCTACCTGCCTGTCCTTGGATTC | pKH3-DTX3L | - | pKH3-DTX3L-DLS/AAA |
| D3-4 | HisD3F:TAAGAAGGAGATATACCATGGCAGAACAAAAAAGCAACTATTTTGAAGTTCCC  HisD3R:GTGGTGGTGGTGGTGCTCGAGTCCTTTTAGAACATACTGCTTCGCCTTTGC | pKH3-DTX3L | pET28a-XhoI/NcoI | pET28a-DTX3L-D34 |
| D1-2 | 28a_DTX3LF:GTTTAACTTTAAGAAGGAGATATACCATGGCCTCCCACCTGCGCCCG  28a_DTX3L_230R:ATCTCAGTGGTGGTGGTGGTGGTGCTCGAGCTTGGTTTCTGGTTCAGAAGGAGAAATGCA | pKH3-DTX3L | pET28a  XhoI/NcoI | pET28a-DTX3L-230 |
| D1-2  T138P | DTX3L_T139PF:GATTCCTGTCTCCAAAAGATCTTTCTTACTGTACCCGCTGACCTGAACTGTAACCTGTTC  DTX3L_T139PR:GAACAGGTTACAGTTCAGGTCAGCGGGTACAGTAAGAAAGATCTTTTGGAGACAGGAATC | pET28a-DTX3L-230 | - | pET28a-DTX3L-230  T138P |
| 14DWC | 28a_DWC2F:GTTTAACTTTAAGAAGGAGATATACCATGGGTGAAAATGTCACGTGTGTG  28a_P14R:ATCTCAGTGGTGGTGGTGGTGGTGCTCGAGTTTTCTAAACGTAATAAGGTAC | pEGFP-PARP14 | pET28a  XhoI/NcoI | pET28a-14DWC |
| 14WC | 28a_14WCF:GTTTAACTTTAAGAAGGAGATATACCATGATAGAATGGCAGTATAATGACAATAAC  28a_P14R:ATCTCAGTGGTGGTGGTGGTGGTGCTCGAGTTTTCTAAACGTAATAAGGTAC | pEGFP-PARP14 | pET28a  XhoI/NcoI | pET28a-14WC |
| 14cat | 28a_14CAT:GTTTAACTTTAAGAAGGAGATATACCATGTGTGTGGTGGAGCTGCTGCCTAG  28a_P14R:ATCTCAGTGGTGGTGGTGGTGGTGCTCGAGTTTTCTAAACGTAATAAGGTAC | pEGFP-PARP14 | pET28a  XhoI/NcoI | pET28a-14cat |

### Table S3: List of the siRNA sequences used in this study (DNA in bold).

| **Dicer siRNA** | **Sense sequence (5**`→ **3**`**)** | **Anti-sense sequence (5**`→ **3**`**)** |
| --- | --- | --- |
| PARP9 13.1 | 5`-CUGUGGCAAAGUCAAUUCUACAA**CA**-3` | 5`-UGUUGUAGAAUUGACUUUGCCACAGGU-3` |
| PARP9 13.2 | 5`-CCAAUGAUGAGUAAUUUGAAAGA**AA**-3` | 5`-UUUCUUUCAAAUUACUCAUCAUUGGCU-3` |
| PARP9 13.3 | 5`-CUGUAAACCCACAUGAUAUUACA**GT**-3` | 5`-ACUGUAAUAUCAUGUGGGUUUACAGAA-3` |
| PARP14 13.1 | 5`-ACUGAGAGCUUGUCCUUAAAAUC**AA**-3` | 5`-UUGAUUUUAAGGACAAGCUCUCAGUGA-3` |
| PARP14 13.2 | 5`-AGCUAUUCUGGCCAAAGAUAAAG**AA**-3` | 5`-UUCUUUAUCUUUGGCCAGAAUAGCUUC-3` |
| PARP14 13.3 | 5`-AGGUCAGAAUUAUCUCUGCAUUU**GA**-3` | 5`-UCAAAUGCAGAGAUAAUUCUGACCUCA-3` |
| DTX3L 13.1 | 5`-GCUACCAAAGUGCAAGCAUGAAU**TC**-3` | 5`-GAAUUCAUGCUUGCACUUUGGUAGCAC-3` |
| DTX3L 13.2 | 5`-AGCAUUGUUUUUCUCUAGAUAAU**TT**-3` | 5`-AAAUUAUCUAGAGAAAAACAAUGCUCA-3` |
| DTX3L 13.3 | 5`-GUGAUUACUUAUUCUAUGAAAGC**AG**-3` | 5`-CUGCUUUCAUAGAAUAAGUAAUCACAA-3` |


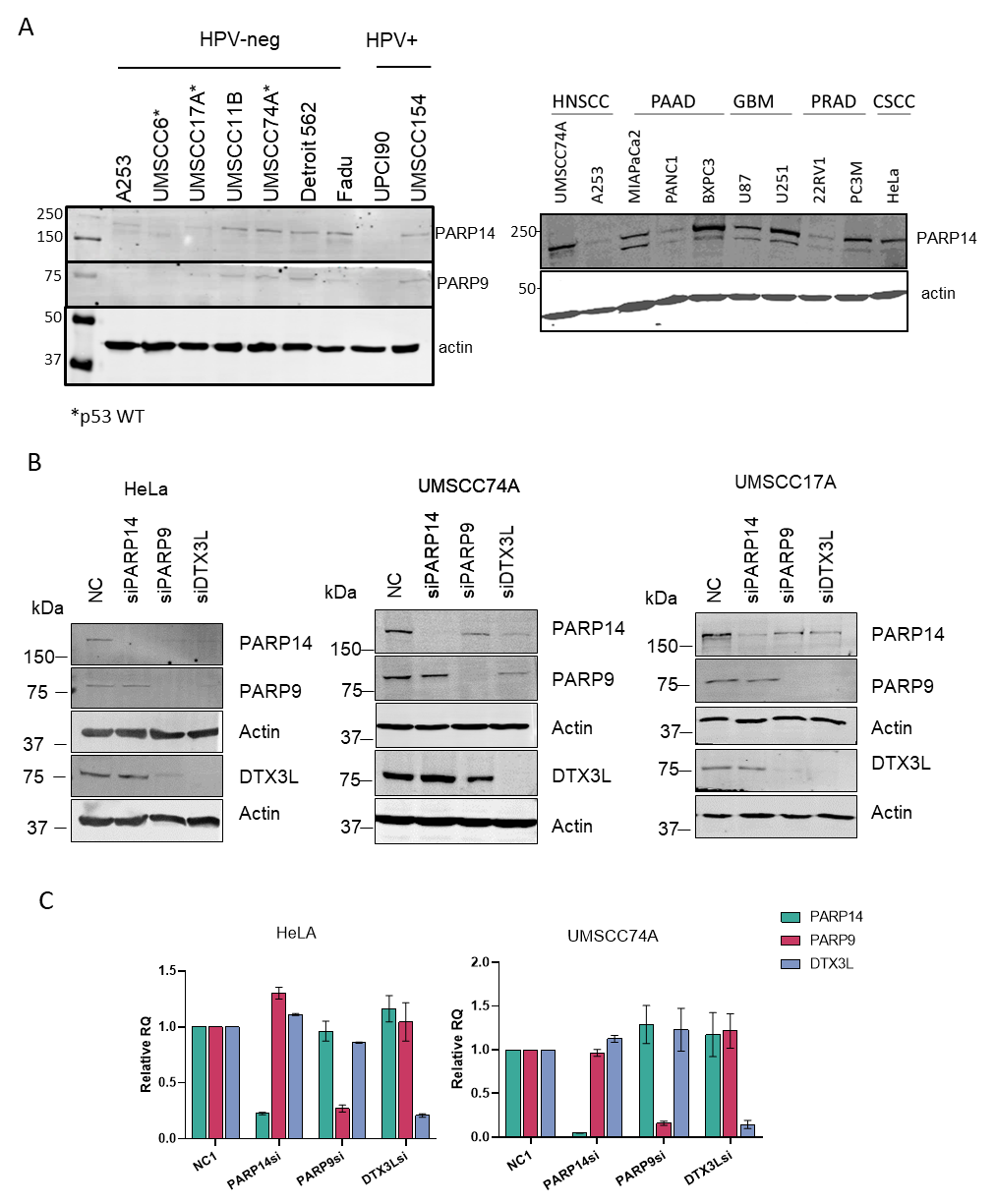


### Figure S1: Regulation of PARP14 levels by PARP9 and DTX3L in other cell lines

A) Immunoblot analysis of a larger panel of HNSCC indicating HPV and TP53 status (left) and comparative levels of PARP14 in a variety of cell lines including HeLa.

B) Immunoblot analysis of PARP14, PARP9 and DTX3L levels 48 h after depletion with siRNA in HeLa, UMSCC74A and UMSCC17A.

C) QPCR analysis of PARP14, PARP9 and DTX3L mRNA levels 24 h after depletion.


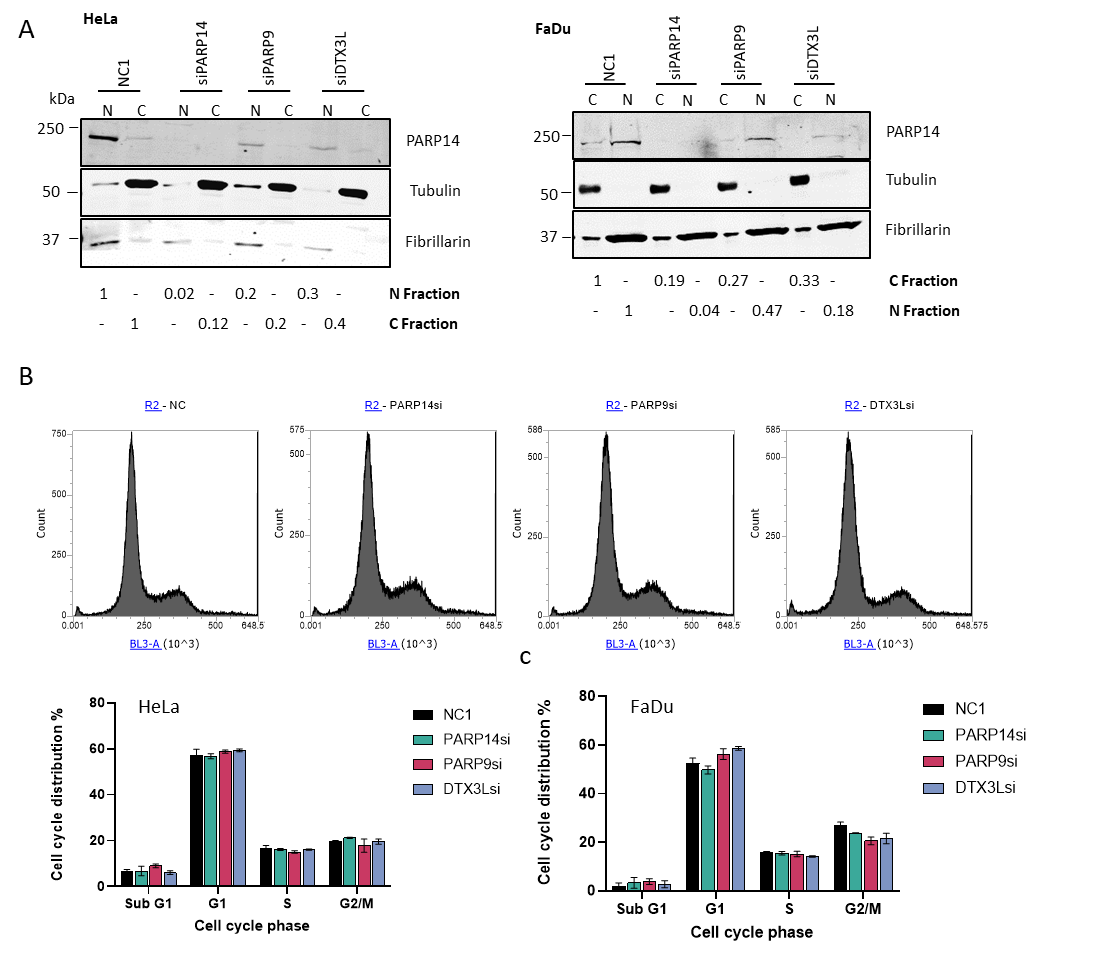


### Figure S2. Consequences of PARP14, PARP9 and DTX3L depletion on PARP14 localisation and cell cycle.

A) Subcellular distribution of PARP14 in cytoplasmic (C) and nuclear (N) fractions in PARP9 and DTX3L depleted cells. HeLa cells transfected with NC1 control or siRNAs targeting PARP9, PARP14, or DTX3L, were harvested 48 h post transfection. Proteins were fractionated and analysed by SDS-PAGE and immunoblotting using Tubulin and Fibrillarin for C and N controls, respectively.

B) Cell cycle analysis of HeLa cells following PARP9, DTX3L and PARP14 depletion. HeLa cells were transfected with NC1 control or siRNA targeting PARP9, DTX3L, or PARP14 for 48 h and harvested for cell cycle analysis. Representative cell cycle profiles of cells transfected with siRNAs against PARP9, DTX3L or PARP14. Graph depicting the percentages of cells in Sub G1, G1, S, or G2/M phases of the cell cycle that is representative of at least two independent experiments with bars presenting means ± SEM.

C) Cell cycle analysis in FaDu 48 h following siRNA treatment.


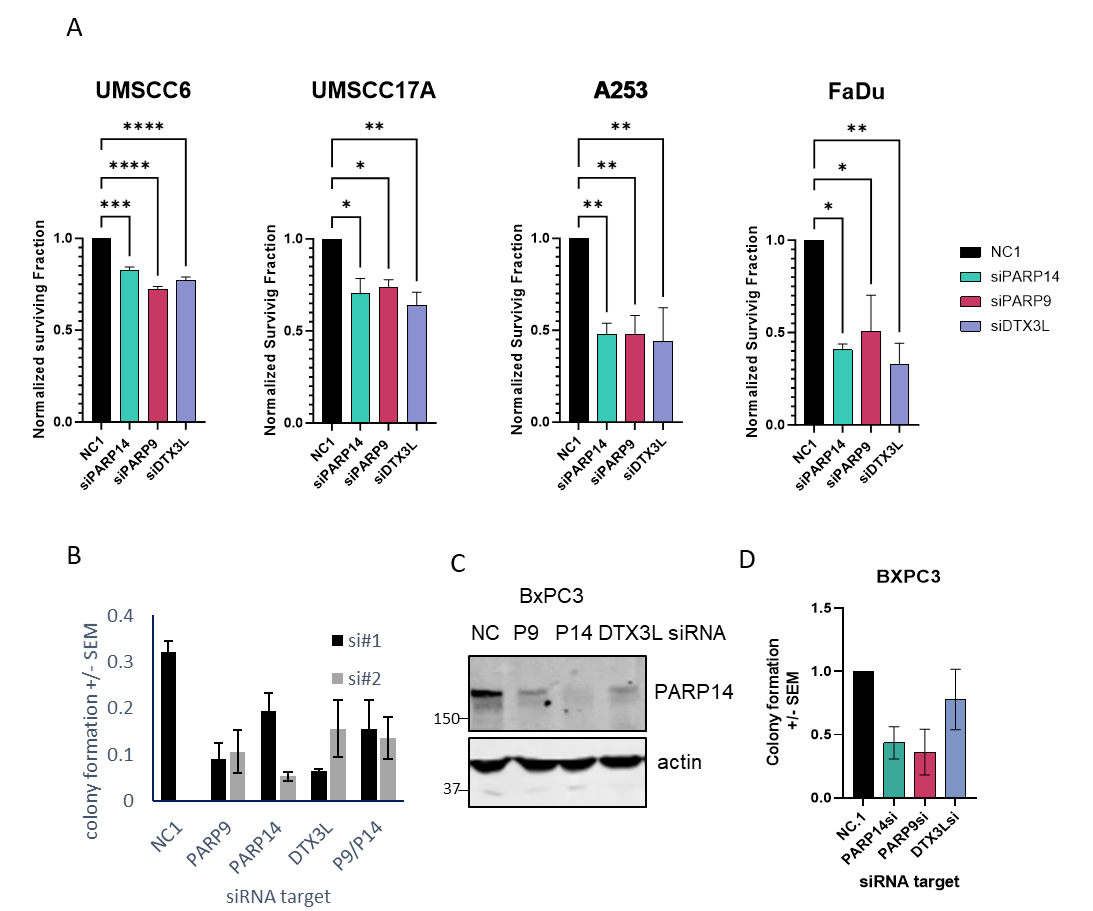


### Figure S3. Colony formation of in other cell lines

A) Colony formation in UMSCC6, UMSCC17A (both TP53 WT) A253 and FaDu (both TP53 mutated) following 48 h siRNA treatment. Statistics were obtained from three replicate experiments (and independent to Figure 2).

B) Colony formation using individual siRNA substrates (PARP9.13.1 and PARP9.13.3 were used since PARP9.13.2 failed to deplete PARP9 in certain cell lines, not shown).

C) Immunoblot to show depletion of PARP14 using siPARP9, siDTX3L and siPARP14 in BXPC3. d) Colony formation of BXPC3 was also reduced following depletion of PARP9, PARP14 and DTX3L, n=3.


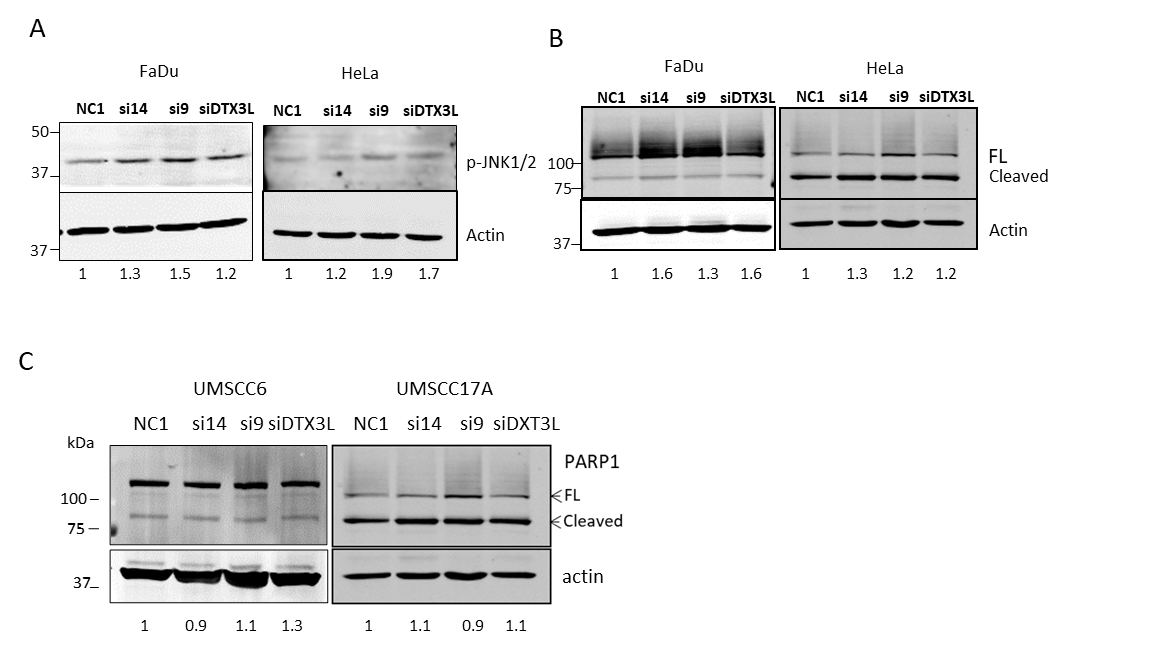


### Figure S4. Markers of apoptosis in PARP14, PARP9 and DTX3L depleted cells.

A) Immunoblot analysis of phosphorylated JNK1 in FaDu and HeLa. Actin was used as a loading control.

B) Immunoblot analysis of cleaved PARP1 in FaDu and HeLa transfected with siRNAs specific to PARP9, PARP14 and DTX3L. Actin was used as a loading control.

C) Cleaved PARP1 levels following knock-down of PARP9, PARP14 and DTX3L in UMSCC6 and UMSCC17A. Values below each lane indicate cleaved PARP1:actin, which were normalised to NC1.


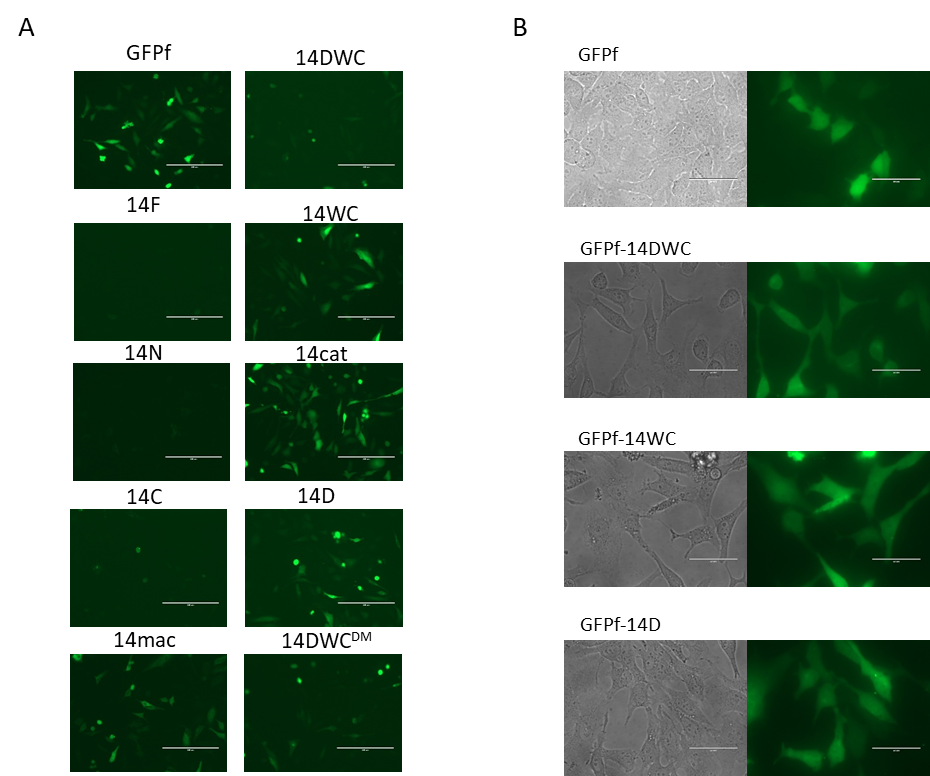


### Figure S5. Expression and localisation of PARP14 truncations in HeLa

A) GFP images of transfections in HeLa (20X magnification) scale bar is 200 mm.

B) Light and GFP images of PARP14 truncations (60X magnification) showing expression in cytoplasmic and nuclear compartments. Scale bar represents 50 mm.


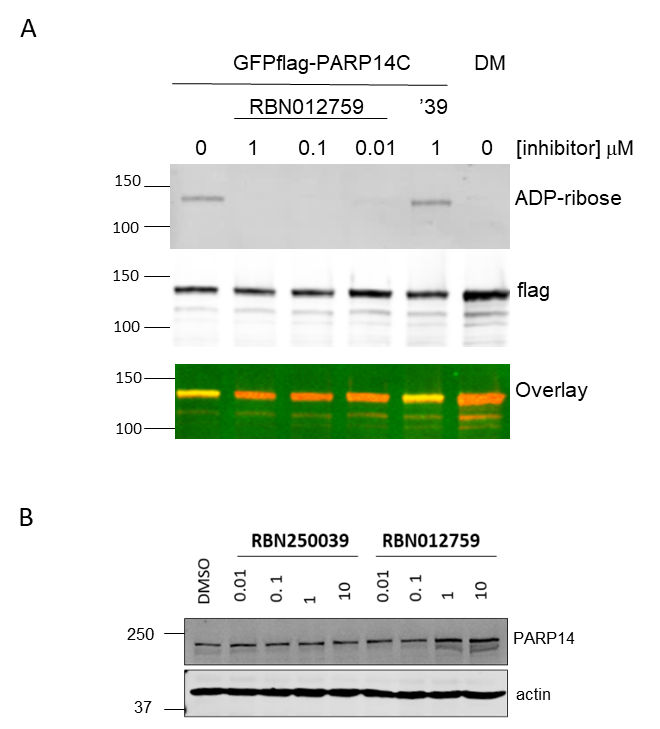


### Figure S6. PARP14 inhibition in cells

A) 14C and a catalytic mutant (DM) was overexpressed in HEK293T. A titration (in μM) of PARP14 inhibitor (RBN012759) and control compound (RBN250039) were added 24 h following transfection and left for a further 24 h. Cells were harvested and analysed by immunoblot with anti-MAR/PAR (CST) and anti-FLAG antibodies.

B) Endogenous PARP14 from FaDu was immunoblotted following 24 h PARP14i treatment. The increase in stability of PARP14 is an indirect effect of PARP14 inhibition observed in Schenkel et al., 2021.


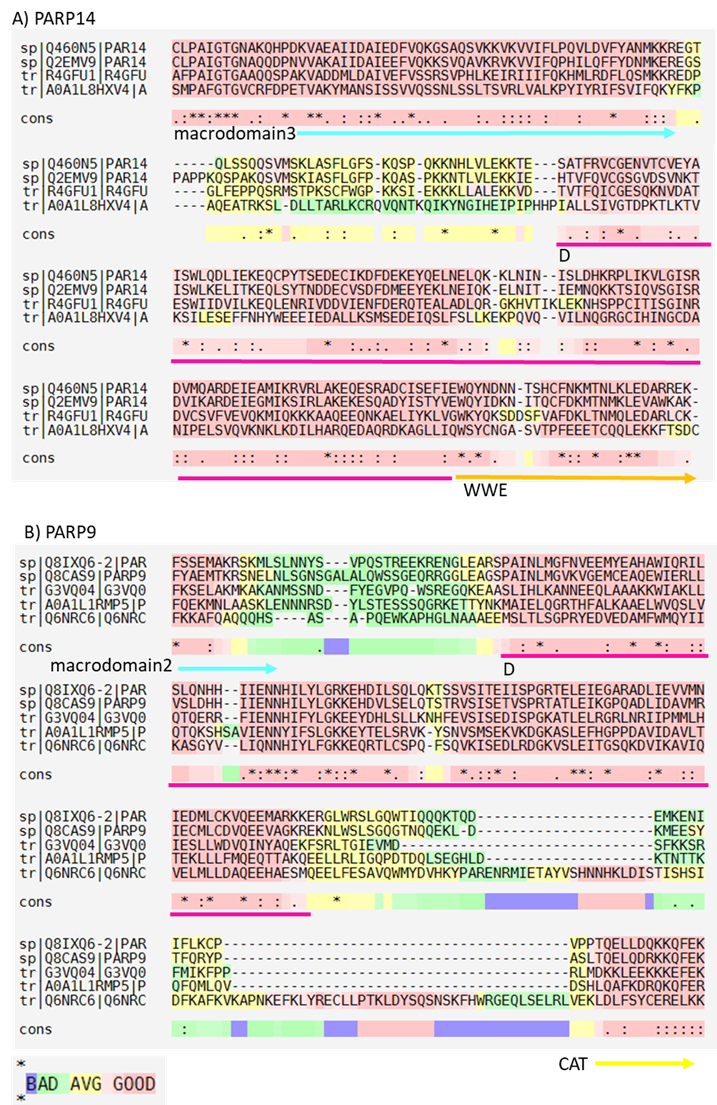


### Figure S7. Alignment and conservation of 9D and 14D

A) A conserved region (D, pink underline) was identified by alignment of Homo sapien, Mus musculus, Gallus gallus and Xenopus laevis sequences by T-coffee alignment tool of PARP14 sequences.

B) A similar region was found in PARP9 species alignments.


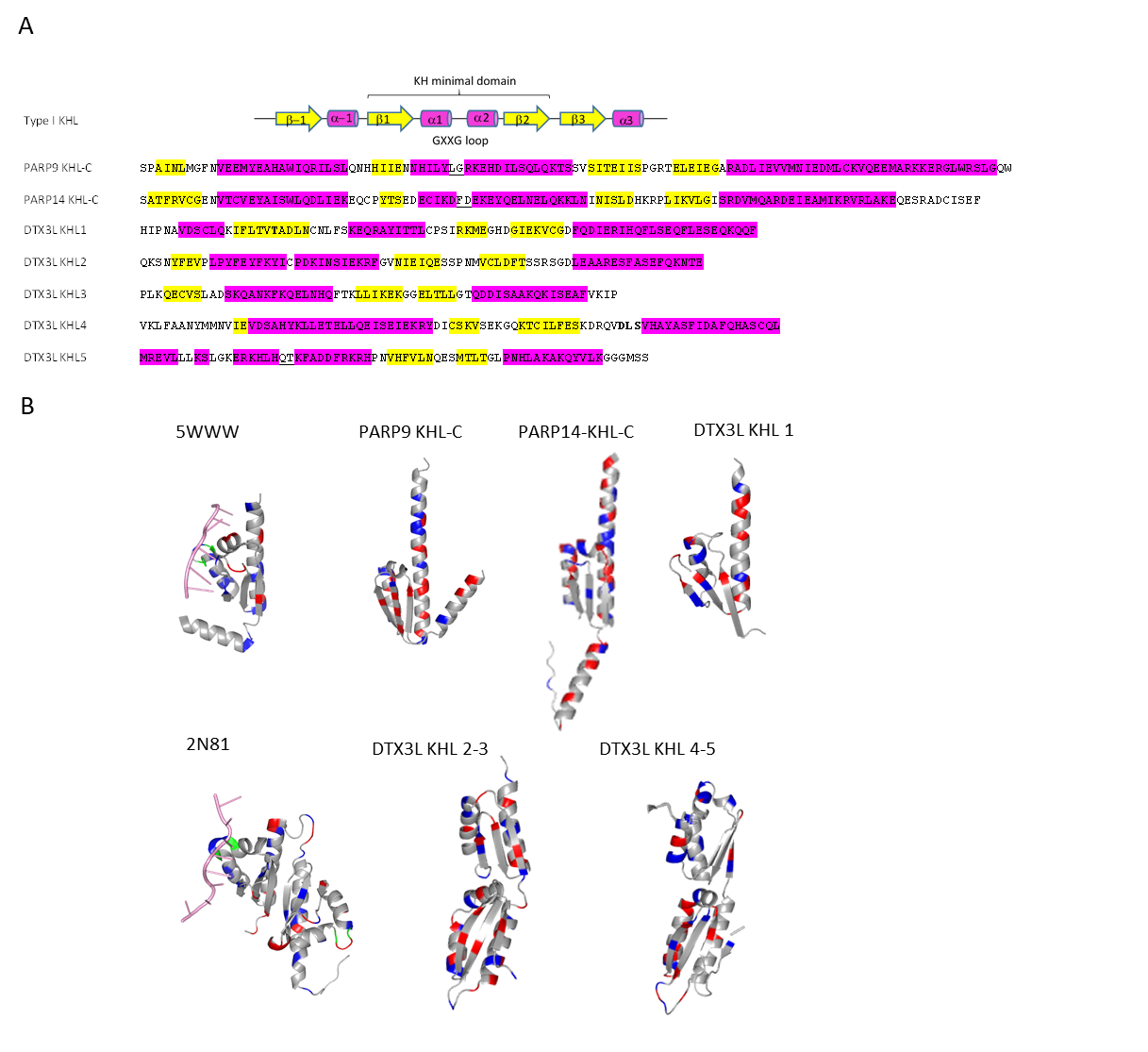


### Figure S8. PSIPRED and AF2 predicted fold of KHL domains of PARP9, PARP14 and DTX3L

A) Predicted secondary structure a-helices (pink) and b-sheet (yellow) of the conserved regions in PARP9 and PARP14 were identical to each other and contained eukaryotic type I KH architecture but did not contain the GXXG loop used in RNA binding. Predicted secondary structure in the DTX3L N-terminal domain were similar to KH domains but varied from the canonical b-a-b-a-a-b-b configuration.

B) AF2 structures of the predicted KHL domains in comparison to PDB structures showing RNA binding. Basic (blue) and acidic (red) residues are highlighted and the GXXG is coloured green. KHL domains were either single (PARP9, PARP14 and DTX3L KHL1) or in tandem as in DTX3L (KHL2-3 and KHL4-5).


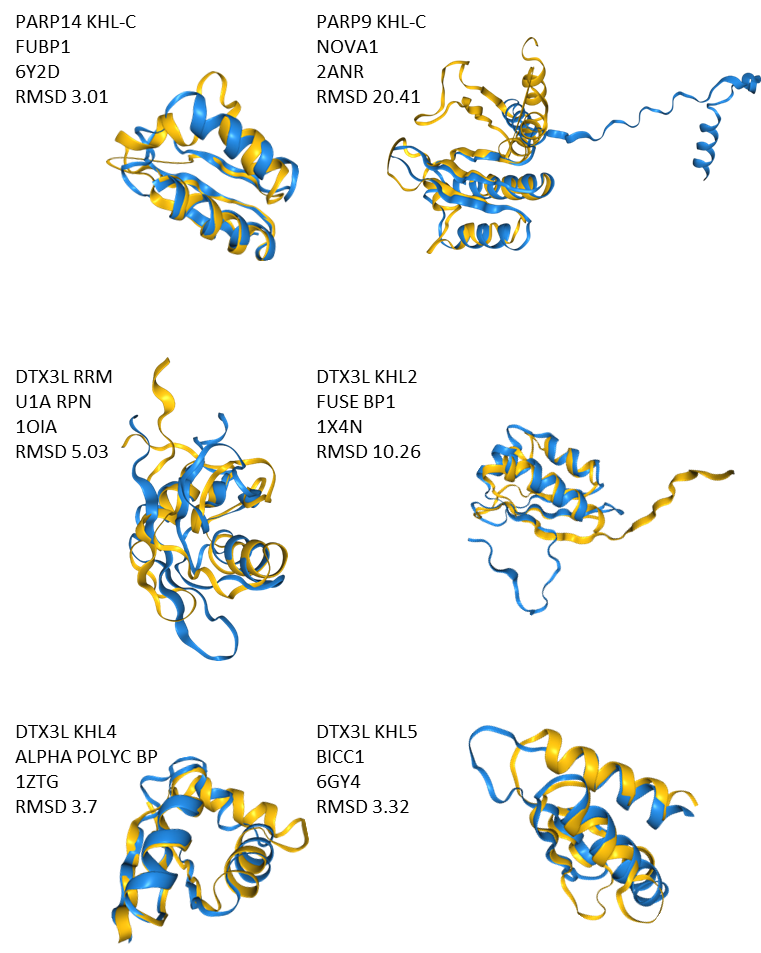


### Figure S9. FoldSeek alignments of closest structural relatives

The AF2 predictions of the PARP14 (C-terminus half), PARP9 and DTX3L (N-terminal domains) were analysed by FoldSeek to identify the closest relative with a PDB structure. No alignment was found for DTX3L KHL1. The deviation of the alignment of the AF2 model (blue) to the PDB structure (gold) was estimated by the root mean square deviation (RMSD) of the polypeptide backbones.


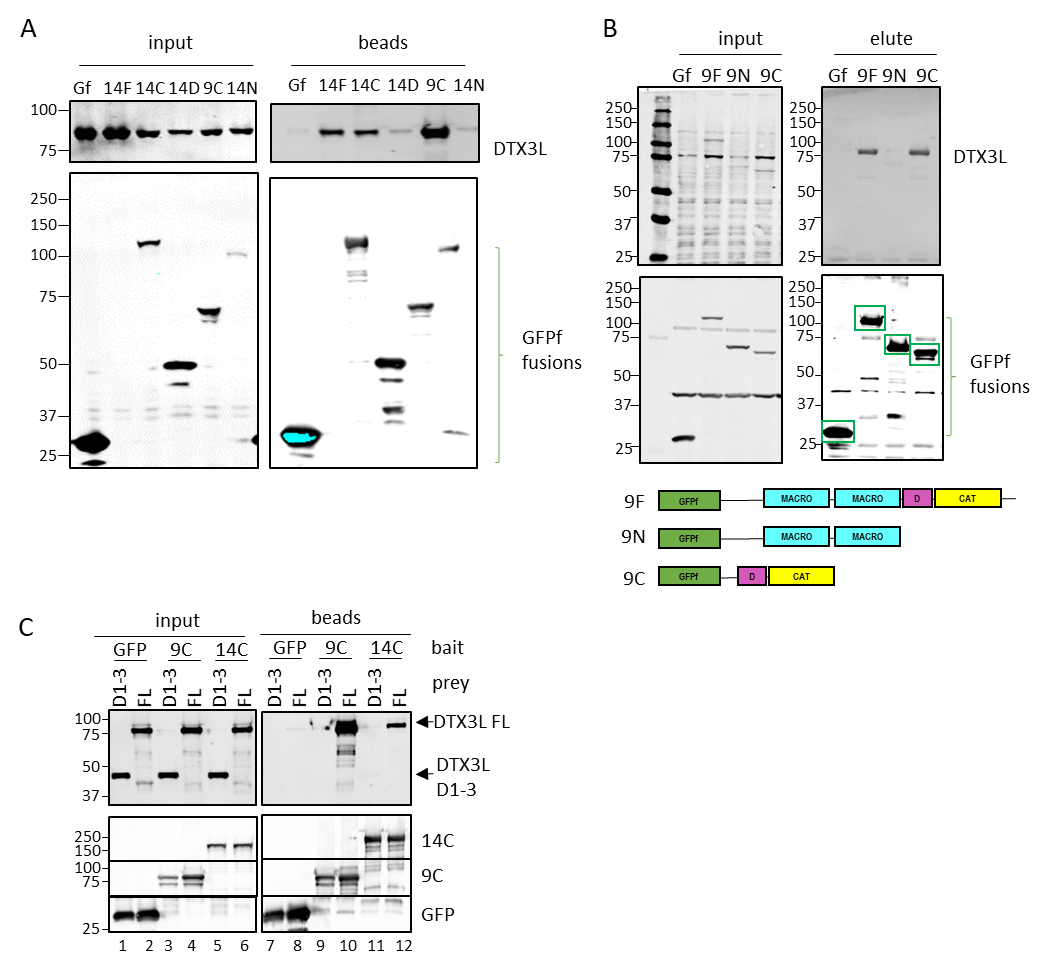


### Figure S10. Minimal domains of DTX3L, PARP9 and PARP14 for interaction

A) GFP-fusion of PARP14 full length and N- and C-terminal halves were used in a Co-IP using overexpressed fusions with untagged DTX3L in HEK293T cells. Similar amounts of DTX3L were detected in the bead fraction with C-terminal half (14C) as the full-length protein (14F). Background quantities of DTX3L were detected in the N-terminal (14N) and KHL domain (14D) similar to GFPflag control. GFP was overexposed to detect 14N.

B) Co-IP of PARP9 truncations in comparison to the full-length protein (9F). DTX3L binding did not require the macrodomains since the C-terminal half (9C) that omits them binds equally as 9F. The levels of DTX3L in the input lane suggest the interaction stabilised protein levels.

C) Anti-FLAG co-IP of GFPf, GFPf-PARP9C and GFPf-PARP14C with DTX3L FL and D1-3 truncation. Inputs and bead elutions were probed with anti-DTX3L (top) and anti-FLAG antibodies (bottom).


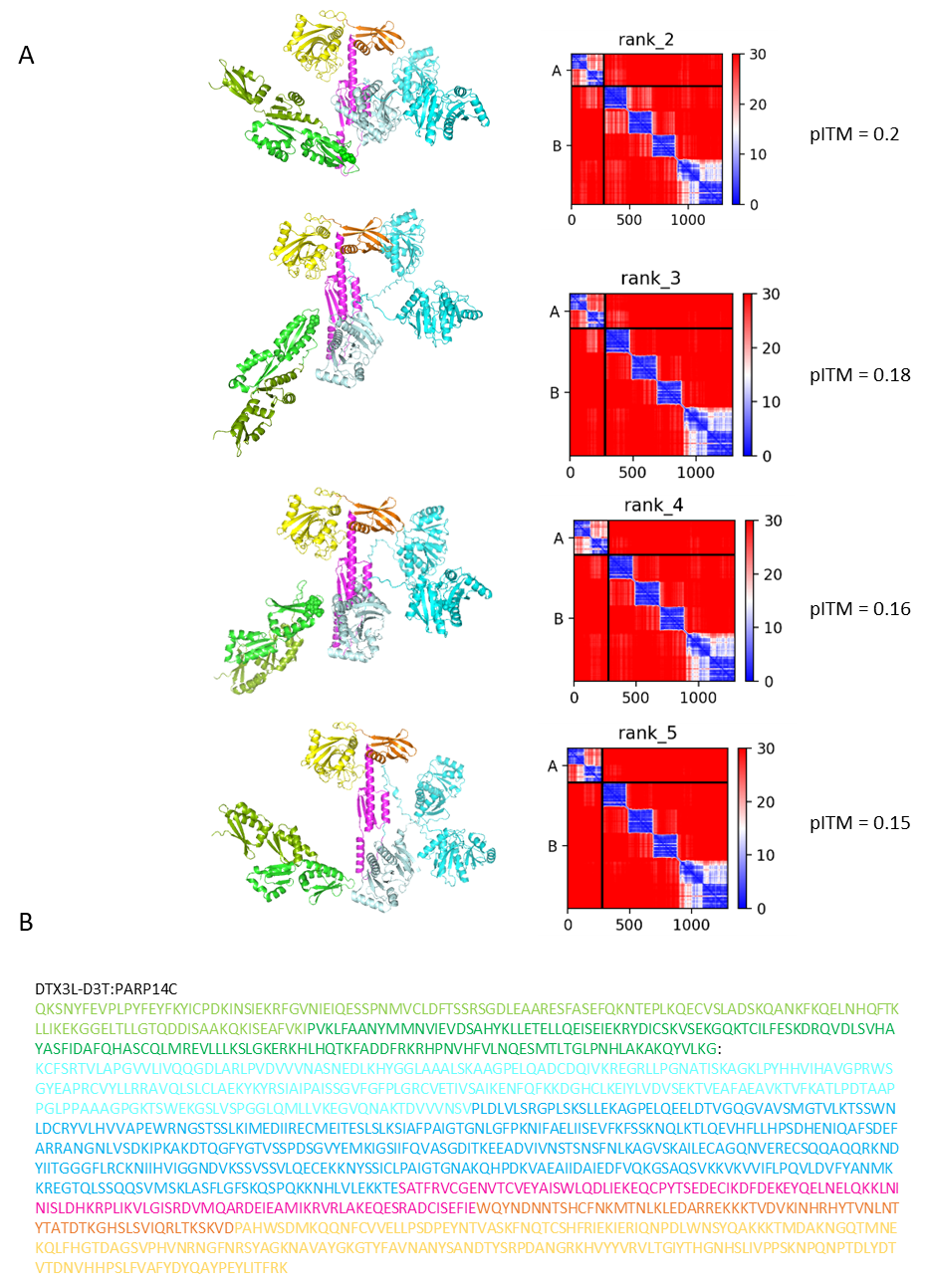


### Figure S11. Other ranked models of PARP14-DTX3L interaction.

A) The predicted interaction of PARP14C with DTX3L KHL2-5 ranks 2-5 show a variation in domain configurations. PAE plots and pITM values of interface indicate a low confidence.

B) Sequence input for interaction predictions colours indicate domains: pale green (DTX3L KLH2-3), green (DTX3L KHL4-5), pale blue (PARP14 macrodomain 1) blue (macrodomain 2-3), pink (KHL-C), Orange (WWE) and gold (catalytic domain).


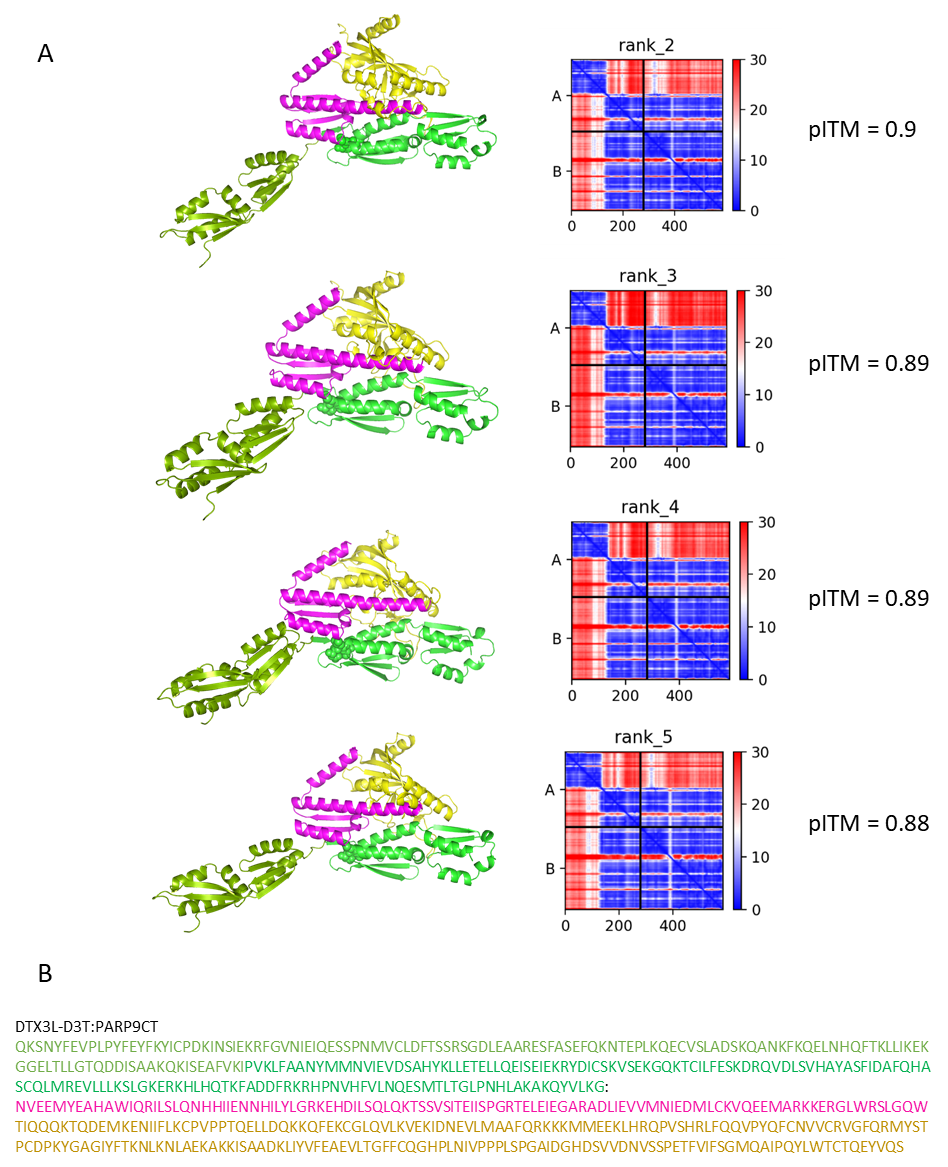


### Figure S12: other ranked models of PARP9-DTX3L interaction

A) The predicted interaction of PARP9C with DTX3L KHL2-5 ranks 2-5 show a variation in domain configurations. PAE plots and pITM values of interface.

B) Sequence input for interaction predictions colours indicate domains: pale green (DTX3L KLH2-3), green (DTX3L KHL4-5), pink (KHL-C), and gold (catalytic domain).


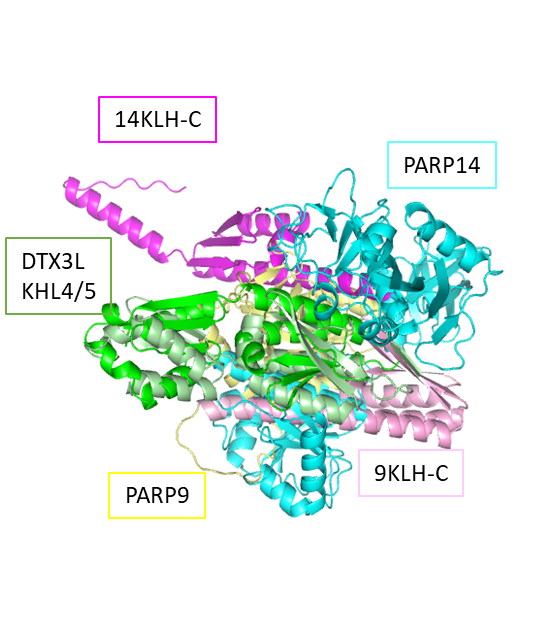


### Figure S13. Superimposing PARP9 and PARP14 interactions on DTX3L KHL4-KHL5

Superposition of PARP14 (cyan):DTX3L(green) and PARP9(yellow):DTX3L(pale green) using aligned DTX3L KHL4 domains shows the KHL domains of PARP14 (magenta) and PARP9 (pink) interact at different positions on the DTX3L KHL4.


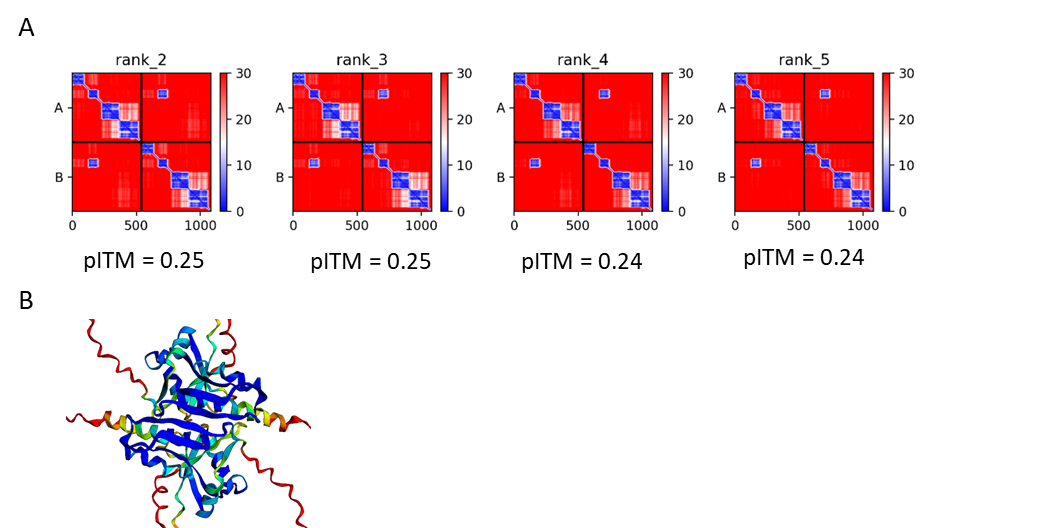


### Figure S14. PAE of other rank models of DTX3L homodimerisation

A) PAE plots of the interaction of N-terminal domains (D1-4) as a homodimer.

B) D1-2 was then used in the homodimerisation prediction and the pLDDT structure of KHL1 is shown where blue = high confidence and red = low confidence.
